# Tri-Layer Composite Nanofiber Wound Dressing Incorporating Glucantime and Silver Nanoparticles for Cutaneous Leishmaniasis Management

**DOI:** 10.3390/jfb17010041

**Published:** 2026-01-15

**Authors:** Hilal Topuz, Murat Inal, Atiye Turker, Zisan Toprak, Emrah Sefik Abamor, Sezen Canim Ates, Serap Acar

**Affiliations:** 1 Department of Bioengineering, Faculty of Chemical and Metallurgical, Yildiz Technical University, Istanbul 34210, Turkey; hilaltopuz95@gmail.com (H.T.); atiye.turker@istinye.edu.tr (A.T.); zisan.toprak@yildiz.edu.tr (Z.T.); esabamor@gmail.com (E.S.A.); 2 Department of Materials and Material Processing Technologies, Kirikkale Vocational School, Kirikkale University, Kirikkale 71450, Turkey; 3 Department of Bioengineering, Faculty of Engineering and Natural Sciences, Kirikkale University, Kirikkale 71450, Turkey; inalmrt@yahoo.com; 4 Department of Medical Services and Techniques, School of Vocational of Healthy, Istinye University, Istanbul 34010, Turkey; 5 Department of Biomedical Engineering, Faculty of Engineering and Architecture, Istanbul Yeni Yuzyil University, Istanbul 34010, Turkey

**Keywords:** cutaneous leishmaniasis, electrospinning, glucantime, nanofiber, silver nanoparticles, wound dressing

## Abstract

Cutaneous leishmaniasis is a zoonotic disease caused by *Leishmania* parasites and leads to chronic, non-healing skin lesions. Although current drugs can control the disease, their use is limited by systemic side effects, low efficacy, and inadequate lesion penetration. Therefore, innovative local delivery systems are required to enhance drug penetration and reduce systemic toxicity. To address these challenges, silver nanoparticles (AgNPs) were synthesized using propolis extract through a green synthesis approach, and a tri-layer wound dressing composed of polyvinyl alcohol and gelatin containing synthesized AgNPs and Glucantime was fabricated by electrospinning. Characterization (SEM-EDX, FTIR, TGA) confirmed uniform morphology, chemical structure, and thermal stability; the wound dressing exhibited hydrophilicity, antioxidant activity, and biphasic release. Biological evaluations against *Leishmania tropica* demonstrated significant antiparasitic activity. Promastigote viability decreased from 76.3% in neat fibers to 31.6% in nanofibers containing AgNPs and 7.9% in tri-layer nanofibers containing both AgNPs and Glucantime. Similarly, the amastigote infection index dropped from 410 in controls to 250 in neat nanofibers, 204 in AgNPs-containing nanofibers, and 22 in tri-layer nanofibers containing AgNPs and Glucantime. The tri-layer nanofibers demonstrated enhanced antileishmanial activity over AgNPs-containing fibers, confirming synergistic efficacy. All nanofibers were biocompatible, supporting their use as a safe platform for cutaneous leishmaniasis treatment.

## 1. Introduction

*Leishmania* is a genus of eukaryotic protozoan intracellular parasites belonging to the family Trypanosomatidae, within the order Trypanosomatida and the class Kinetoplastea. It includes nearly 20 species that are transmitted by female sandflies (of which there are about 90 species), leading to the disease known as leishmaniasis [1]. It causes leishmaniasis by infecting host immune cells, primarily macrophages, in tissues such as the skin, liver, and spleen [2]. The disease is endemic in nearly 100 countries, and an estimated 350 million individuals are at risk. It affects approximately 12 million people, with around 2 million new cases reported annually [3]. Aside from malaria, leishmaniasis is the most fatal parasitic disease globally [4].

Leishmaniasis can be classified into three primary forms: visceral leishmaniasis (also known as kala-azar), cutaneous leishmaniasis, and mucocutaneous leishmaniasis.

Cutaneous leishmaniasis (CL) is a major public health concern worldwide. The World Health Organization (WHO) classifies CL as both an emerging and uncontrolled condition, as well as a neglected tropical disease [5].

Despite the widespread use of pentavalent antimonials, amphotericin B, and miltefosine as standard treatments for cutaneous leishmaniasis, these therapies have significant limitations. Drugs used to treat CL often fail to penetrate skin lesions effectively, and pentavalent antimonials, such as meglumine antimoniate, have been reported to cause various systemic adverse effects, including liver and kidney injury as well as irregularities in cardiac rhythm, and these complications can jeopardize patient safety and restrict their continued therapeutic application. Although Amphotericin B is an effective agent, its use is limited by pronounced nephrotoxicity and frequent infusion-related reactions, including fever, chills, and hypotension. Moreover, miltefosine, recognized as the first oral therapeutic approved for leishmaniasis, faces important limitations due to its high cost and its established teratogenicity, factors that prohibit its use in pregnant women and markedly restrict its availability in regions with limited healthcare resources [6,7].

In recent years, the side effects associated with synthetic drugs, along with their medical and economic consequences, have increased the interest in herbal and other natural resources, as well as in nanotechnological approaches, for therapeutic purposes. Eco-friendly, non-toxic, nano-sized metal-based nanoparticles (NPs) can be synthesized using natural extracts. With this method, known as green synthesis, NPs are obtained easily and ecologically without the need for high pressure, high temperature values and toxic chemicals [8].

Due to their unique size, shape, and structure, silver nanoparticles (AgNPs) can interact with cell membranes and inhibit pathogen proliferation. AgNPs exhibit broad-spectrum antimicrobial activity against bacteria, fungi, and viruses [9,10]. AgNPs, owing to their nanoscale size, are internalized mainly via endocytosis and they act by disrupting membranes, binding to cellular thiol groups, generating ROS, damaging intracellular constituents, and inhibiting respiratory enzymes [11]. Propolis, a resinous bee product rich in flavonoids and phenolic acids as attracted attention for its potent antioxidant, antibacterial, and antileishmanial properties [12]. These properties make propolis particularly relevant for wound-related and antileishmanial applications. Several studies have demonstrated that propolis exhibits pronounced wound-healing activity by promoting re-epithelialization, enhancing epithelial cell proliferation and activation, reducing oxidative stress, and stimulating collagen synthesis, thereby accelerating tissue repair and extracellular matrix remodeling [13,14]. Importantly, increasing evidence supports the antiprotozoal and antileishmanial activity of propolis. Machado et al. [15] reported that the IC_50_ values of propolis extracts from Brazil and Bulgaria against *L. major*, *L. braziliensis*, *L. amazonensis* and *L. chagasi* ranged from 2.8 to 229.3 µg/mL after 24 h of incubation, and that these extracts showed antileishmanial activity.

Ozbilge et al. [16] reported that ethanolic propolis extract obtained from Kayseri showed antileishmanial effect against *Leishmania tropica* from a concentration of 32 µg/mL and that the efficacy increased with increasing concentration and incubation time. Further supporting these findings, Aksoy et al. [17] reported that propolis showed a stronger antileishmaniasis effect on *L. tropica* promastigotes compared to honey species, and that the IC_50_ value was 82.98 µg/mL after 48 h. Accordingly, our AgNPs were produced by a propolis-mediated green synthesis, with propolis acting as both the reducing and capping agent.

Wound dressings play a central role in wound-care research and clinical practice worldwide. Electrospun fibers, which closely mimic the extracellular matrix and provide high porosity with a large surface-area-to-volume ratio, have been widely utilized in biomedical applications, including antibacterial meshes, hemostatic mats, wound dressings, and drug-delivery systems [18]. Since cutaneous leishmaniasis manifests as dermal lesions, topical or transdermal delivery of therapeutic agents to the lesion site is particularly advantageous. Among current strategies, electrospun nanofibers have emerged as promising carriers for localized therapy. For instance, Rahimi et al. [19] fabricated chitosan/poly (ethylene oxide)/berberine nanofibers via electrospinning and reported potent in vitro leishmanicidal activity against *Leishmania major* in both promastigote and amastigote stages. Bahraminegad et al. [20] developed polylactic acid-chitosan nanofibers decorated with amphotricin B for the treatment of cutaneous leishmaniasis wounds and investigated the antileishmanial activity of these systems against *Leishmania major* promastigotes. The study reported that the drug-loaded nanofibers provided higher parasite inhibition on promastigotes compared to free amphotericin B.

Nanofibers are produced from a wide range of natural and synthetic polymers. Gelatin, derived from the hydrolysis of collagen, is a highly biocompatible and biodegradable natural polymer. Its intrinsic arginine–glycine–aspartic acid (RGD) motifs promote cell adhesion to material surfaces. Moreover, gelatin is rich in hydroxyproline, glycine, and proline amino acids known to facilitate and accelerate wound healing [21]. Blending multiple polymers is crucial for exploiting their complementary properties and producing wound dressings that are both mechanically compliant and functionally effective. Gelatin, as a denatured collagen derivative, exhibits low antigenicity, high hydrophilicity, and intrinsic cell-adhesive motifs; however, its standalone use is limited due to insufficient mechanical strength, poor thermal stability, and rapid degradation [22].

In recent years, poly (vinyl alcohol) (PVA) has attracted considerable interest in dermatologic and wound healing applications owing to its biocompatibility, hydrophilicity, biodegradability, and excellent film-forming capacity; it is widely used in gel-based dressings and medical cottons, and electrospun PVA fibers display robust mechanical performance [23,24]. Notably, PVA can compensate for gelatin’s low mechanical strength and impart water resistance and long-term stability to the construct, whereas gelatin enhances biocompatibility, an effect corroborated in antibacterial PVA/gelatin nanofiber systems [25]. To achieve controlled drug release, more advanced electrospinning strategies, such as coaxial, triaxial, and multilayer “sandwich” architectures are employed to tune release kinetics, thereby enabling sustained delivery, improved drug utilization, and reduced side effects [22]. For example, in a sandwich-type wound dressing, an outer PCL nanofiber layer can deter microbial adhesion, a middle PCL/gelatin layer incorporating AgNPs provides antimicrobial activity, and an inner PCL/gelatin layer promotes cell adhesion and accelerates healing. Combining gelatin with PCL increases biocompatibility while PCL contributes mechanical reinforcement and moderates AgNP release, mitigating cytotoxicity [21]. Along similar lines, Asgari et al. designed core–shell nanofibers to minimize the adverse effects of amphotericin B, using a PVA–chitosan–amphotericin B core and a PEO–gelatin shell; the fibers were comprehensively characterized, were non-cytotoxic, and achieved up to 84% killing of *Leishmania major* promastigotes in vitro, supporting their promise as a topical delivery platform for CL [26].

Wound dressings and electrospun nanofiber membranes containing silver nanoparticles are being extensively researched in the field of wound healing due to their strong antimicrobial properties, large surface area, and the advantage of being able to be applied directly to the wound site; in particular, the potential of these systems to reduce the risk of chronic infections by inhibiting bacterial growth is noteworthy [27,28]. These systems typically focus on general antimicrobial activity rather than addressing disease-specific therapeutic requirements. Although several studies have reported the antiparasitic potential of AgNPs [29,30] or natural products [31,32] against *Leishmania* species, their integration into electrospun wound dressings specifically designed for cutaneous leishmaniasis remains largely unexplored. Moreover, most reported electrospun systems are based on single-layer architectures or single therapeutic agents, which may limit their ability to achieve controlled release profiles and sustained local efficacy.

We therefore propose a tri-layer electrospun nanofiber dressing for cutaneous leishmaniasis. The construct comprises (i) a thin PVA contact layer to maintain moisture and conformability, (ii) a gelatin layer incorporating Glucantime, which promotes cell adhesion and facilitates the release of the antileishmanial agent, and (iii) an outer PVA layer incorporating green-synthesized AgNPs, which enable extended release and reinforce therapeutic efficacy against leishmanial parasites. These composite nanofibers were prepared and subsequently their antileishmanial efficacy was evaluated by in vitro experiments in the culture of promastigote and amastigote forms of *Leishmania tropica* parasite, causative agent of CL, to recommend a new biotechnological treatment approach against this neglected tropical disease.

## 2. Materials and Methods

### 2.1. Materials

Poly (vinyl alcohol) (M_w_ = 145,000 Da) and gelatin (Type A, from porcine skin, 175 bloom) were purchased from Sigma-Aldrich (Darmstadt, Germany). Propolis (powder form) was purchased from a local market in Turkey (Erzurum). Glucantime was purchased from Kwality Pharmaceuticals Ltd. (Nag Kalan, Amritsar, Punjab, India). The L929 mouse fibroblast cell line (ATCC^®^ CCL-1™), the J774 mouse macrophage cell line (ATCC-TIB-67) and the *Leishmania tropica* strain (MHOM/TR/99/EP39) was obtained from (Dr. Emrah Sefik ABAMOR), Yıldız Technical University Cell Culture Laboratory.

### 2.2. Green Synthesis and Characterization of AgNPs

Silver nanoparticles (AgNPs) were synthesized using a green synthesis approach based on the reducing and stabilizing properties of propolis [33]. For this purpose, an aqueous extract of commercial propolis powder was first prepared. Briefly, 2.5 g of propolis powder was dispersed in 25 mL of distilled water and sonicated in an ultrasonic bath (Bandelin- Sonorex Digital 10P, Berlin, Germany) at 45 °C for 30 min to obtain the aqueous extract [34]. The resulting extract was first passed through coarse filter paper to remove large particulate matter. The filtrate was then centrifuged at 9000 rpm for 40 min to eliminate remaining insoluble residues. Subsequently, the supernatant was filtered through a syringe filter with a pore size of 0.45 µm to obtain a clear aqueous extract. This purified extract was then used directly in the green synthesis of silver nanoparticles [35]. To synthesize the AgNPs, different volumes (0.5, 1, 2, 5 and 10 mL) of the aqueous propolis extract were mixed with a 1 mM silver nitrate (AgNO_3_) solution. The total reaction volume in each case was adjusted to 20 mL. The pH of the reaction medium was systematically adjusted to 7.5, 9.5 and 11.5 using NaOH solutions to investigate the effect of pH (HANNA Instruments HI 2211 pH/ORP Meter, Woonsocket, RI, USA) on nanoparticle formation. Furthermore, the reactions were conducted at various temperatures (40 °C, 60 °C, and 80 °C) to examine the influence of temperature on the synthesis efficiency and particle characteristics. Following the reaction, the synthesized AgNPs were filtered through a 0.45 µm membrane then stored at +4 °C for further characterization. The synthesized nanoparticles were characterized comprehensively to determine their optical, morphological, structural, and surface properties.

UV-Vis spectroscopy: The optical properties of AgNPs were evaluated by recording their absorption spectra in the range of 200–800 nm using a UV-Vis spectrophotometer (UV-1700 Pharmaspec, Shimadzu, Kyoto, Japan) [36]. The presence of the characteristic surface plasmon resonance (SPR) peak confirmed nanoparticle formation.

Transmission electron microscopy: For morphological analysis a drop of the processed sample was then placed onto a carbon-coated copper grid or carbon tape and allowed to dry under ambient conditions. Imaging was carried out using a transmission electron microscope (FEI, Tecnai G^2^ Spirit Biotwin CTEM, Hillsboro, OR, USA) (METU Central Laboratory), providing detailed information on particle size and shape [36].

Average particle size, polydispersity index (PDI), and zeta potential analysis: The average hydrodynamic diameter and PDI of the synthesized silver nanoparticles were determined using a Zetasizer Nano ZS (Malvern Instruments Ltd., Malvern, UK), which operates based on photon correlation spectroscopy [37]. The instrument is equipped with a He–Ne laser (λ = 633 nm, power = 4 mW). Measurements were carried out at 25 ± 0.1 °C, using water as the dispersant with a viscosity of 0.8872 cP and a refractive index of 1.330. Prior to measurement, nanoparticle suspensions were diluted at a 1:30 ratio with deionized water to avoid multiple scattering effects. Each sample was analyzed in triplicate to ensure reproducibility, and measurement durations as well as voltage settings were automatically adjusted by the instrument’s software (version 7.1). Zeta potential (ζ) values were also measured using the same instrument via electrophoretic light scattering (ELS) to evaluate the surface charge and colloidal stability of the nanoparticles. The measurements were performed under the same temperature (25 ± 0.1 °C) and viscosity conditions, with a dielectric constant of 79 and a Smoluchowski model parameter (κa) set to 1.50. Zeta potential analyses were also conducted in triplicate under identical dilution conditions to maintain consistency across all measurements [37].

Fourier transform infrared spectroscopy (FTIR) analysis: To identify the functional groups involved in the reduction and stabilization of AgNPs, FTIR spectra were recorded in the range of 4000–400 cm^−1^ using an FTIR spectrometer (Shimadzu, Kyoto, Japan) [38]. The spectra of both the pure aqueous propolis extract and the synthesized nanoparticles were compared to determine the characteristic bands responsible for capping and stabilization.

### 2.3. Fabrication of Nanofibers

For production of nanofibers via electrospinning, polyvinyl alcohol (stock solution of PVA, 12% (*w*/*v*) in ultrapure water) [39] and gelatin (Gel, 30% (*w*/*v*) in formic acid) [40] solutions were prepared separately. The PVA mixture was heated to 90 °C and stirred continuously using a magnetic stirrer for 4 h until a clear and homogeneous solution was achieved. Gelatin solution was prepared under magnetic stirring at room temperature for 0.75 h, ensuring complete dissolution. To provide therapeutic and antileishmanial activity, Glucantime (Glu) was incorporated into the gelatin solution at concentrations of 1.2%, 2.4%, and 3.6% (*w*/*w*, relative to gelatin weight). These concentrations were selected to represent low, medium, and high drug-loading levels within a range suitable for electrospinning of gelatin solutions. Stepwise concentration increments are commonly employed in antileishmanial drug delivery studies to investigate dose response trends [41], while maintaining suitability for polymer-based processing, consistent with approaches reported in the literature [42,43].

Separately, AgNP solution incorporation ratio was fixed at 20% (*v*/*v*) based on re-ported protocols in the literature [44,45,46]. Consequently, the PVA solution was diluted to 9% (*w*/*v*). The AgNPs added to PVA solution was then homogenized by magnetic stirring for 20 min to ensure uniform dispersion. Although AgNPs can display cytotoxicity in free solution, their behavior may differ when incorporated into a polymeric matrix, and this concentration was chosen based on its compatibility with our formulation conditions and electrospinning process. The loading amounts of Glu and AgNPs were determined according to the overall design of the study. The AgNP content was kept constant in all formulations, while three different Glu levels were used to produce dressings with low, medium, and high drug loading. This formulation strategy enabled uniform incorporation of the components into the polymer matrix and a systematic comparison of Glu-dependent biological responses under identical AgNP conditions.

Tri-layered nanofiber wound dressings were fabricated using a horizontal electrospinning setup equipped with a stainless-steel needle (21G, inner diameter 0.51 mm). The system consisted of a high-voltage power supply (PHYWE, Göttingen, Germany), programmable syringe pumps (NE-1000; New Era Pump Systems Inc., Farmingdale, NY, USA), and a flat aluminum collector. Electrospinning was carried out sequentially for each layer, with specific voltage and flow rate parameters optimized for each polymer solution. Throughout the process, the needle-to-collector distance was maintained at a constant 15 cm. All electrospinning steps were performed under ambient laboratory conditions.

The inner layer, designed for direct contact with the wound surface, was composed of pure PVA nanofibers and was electrospun at 15 kV with a flow rate of 0.9 mL/h (PVA, 9% *w*/*v*). The middle layer, containing gelatin nanofibers loaded with Glu at different concentrations, was fabricated under a voltage of 18 kV and a flow rate of 0.48 mL/h. The outermost layer, consisting of PVA nanofibers incorporating AgNPs, was produced at 17 kV with a flow rate of 0.6 mL/h. The nanofibers were collected onto a stationary flat collector covered with aluminum foil to facilitate the successive deposition of each layer and formation of the final multilayer structure. As summarized in Table 1, a total of nine different nanofiber formulations were fabricated.

### 2.4. Physicochemical Characterization of Tri-Layer Composite Nanofiber Wound Dressing

#### 2.4.1. Scanning Electron Microscopy–Energy-Dispersive X-Ray Spectroscopy (SEM–EDX)

The surface morphology of the electrospun nanofiber mats (PVA, PVA-AgNPs, Gel, Gel-Glu3.6% and PVA-AgNPs/Gel-Glu3.6%/PVA nanofibers with dimensions of 1 × 1 cm^2^ was examined using a scanning electron microscope (Zeiss EVO LS 10) [47]. Prior to imaging, the samples were sputter-coated with a thin layer of Au/Pd using a sputter coater (SC7640, Polaron Equipment Ltd., Watford, UK) to enhance surface conductivity and prevent charging during SEM observation. SEM imaging was performed at an accelerating voltage of 3.00 kV under high-vacuum conditions.

Elemental analysis was carried out using energy-dispersive X-ray spectroscopy (EDX) attached to the SEM system to determine the presence of silver (Ag) and antimony (Sb) within the nanofiber structures. EDX point analysis was employed for the elemental detection of Ag, attributed to AgNPs incorporated into the PVA-based outer layer, and Sb, originating from Glucantime in the gelatin-based middle layer, to determine the elemental composition of the nanofiber structures.

Fiber diameter distributions were quantified by analyzing SEM micrographs using ImageJ software (version 1.53, National Institutes of Health, Bethesda, MD, USA). At least 100 individual fiber diameters were measured per sample to ensure statistical reliability, and the average fiber diameter and standard deviation were calculated accordingly.

#### 2.4.2. FTIR Analysis

FTIR spectroscopy was employed to analyze the chemical structure of the polymers used in the wound dressing material, as well as the functional groups of AgNPs and the incorporated drug. For this purpose, FTIR spectra of AgNPs, Glucantime, PVA nanofibers, PVA-AgNPs nanofibers, Gel, Gel-Glu3.6% nanofibers were studied. The nanofibers samples cut in 1 × 1 cm^2^ dimensions. The measurements were performed in attenuated total reflectance (ATR) mode within the wavenumber range of 4000–400 cm^−1^, with a resolution of 4 cm^−1^ and 16 scans for each spectrum, using an FTIR spectrometer (Shimadzu IRTracer^TM^-100, Kyoto, Japan; METU Central Laboratory) [48]. All spectra were baseline corrected and normalized prior to further analysis.

#### 2.4.3. Thermogravimetric Analysis (TGA)

TGA was performed to evaluate the thermal stability and decomposition behavior of the nanofiber wound dressing samples. For this purpose, AgNPs, Glucantime, PVA nanofibers, PVA-AgNPs nanofibers, Gel nanofibers, Gel-Glu3.6% nanofibers, and PVA-AgNPs/Gel-Glu3.6%/PVA nanofibers were analyzed using a DSC/TGA instrument (TA Instruments SDT 650 Simultane DSC/TGA, New Castle, DE, USA; METU Central Laboratory) under a nitrogen atmosphere to prevent oxidative degradation. Approximately 10 mg of each sample was placed in a platinum pan and heated from 25 °C to 900 °C at a constant heating rate of 10 °C/min [48]. The weight loss was recorded as a function of temperature to determine thermal degradation stages and residue content.

#### 2.4.4. Contact Angle Analysis

The surface wettability of PVA and PVA-AgNPs nanofibers (2 × 2 cm^2^) was determined using water contact angle (WCA) measurements with the sessile drop method. A 5 µL droplet of distilled water was placed on the sample surface at room temperature. The droplet image was recorded by a CCD camera integrated into the Attension Theta Lite Optical Tensiometer (Biolin Scientific, Espoo, Finland) [49]. The contact angle was calculated using the instrument software (version 4.2.0 (r10009)). Measurements were performed at three different points per sample, and the mean value was recorded.

#### 2.4.5. Water Absorption Capacity (WAC)

WAC of the nanofiber samples was determined according to the ASTM D-570 standard [50]. Prior to testing, the samples were cut into 2 × 2 cm^2^ pieces, and their initial dry weights (W_0_) were recorded. The specimens were then immersed in an adequate amount of deionized water to ensure complete submersion and kept at 25 °C for 24 h. After immersion, excess surface water was gently removed using filter paper, and the samples were reweighed (*Wₛ*) [51]. The water absorption capacity was calculated using the following Equation (1):

(1) $$WAC\;(\%)=\frac{W_{s}-W_{0}}{W_{0}}\times 100$$

where *Wₛ* is the swollen weight and *W*_0_ is the initial dry weight of the sample.

The water absorption capacity measurements were performed in triplicate, and the results are presented as mean ± standard deviation (SD).

#### 2.4.6. In Vitro Release Profile of Glucantime and Silver Nanoparticles

Prior to the release study, the silver and Glucantime content of the PVA-AgNPs/Gel-Glu3.6%/PVA nanofiber was quantified using inductively coupled plasma mass spectrometry (Agilent 7700X ICP-MS Systems, Agilent Technologies, Santa Clara, CA, USA) after microwave-assisted acid digestion method. For sample preparation, 10 mg nanofiber were placed in reaction vessels containing 10 mL of 65%, HNO_3_ and the sample burned with microwave digestion unit (MARS 6 iWave System, CEM, Matthews, NC, USA). The collected solution was subsequently analyzed by ICP-MS to determine the Glucantime (based on the elemental antimony concentration) and silver content of the nanofiber [52].

To evaluate the Glu and silver nanoparticle release, the nanofiber (10 mg, PVA-AgNPs/Gel-Glu3.6%/PVA) were incubated in 5 mL of phosphate-buffered saline (PBS, pH 7.4). The samples were maintained at 37 °C in a shaker incubator to simulate physiological conditions. At predetermined intervals (0.25, 0.5, 0.75, 1, 2, 4, 6, 8, 24, 48, and 72 h), the entire release medium was removed and replaced with an equal volume of freshly prepared PBS. The collected solutions were subsequently analyzed by ICP-MS to determine the concentrations of Glucantime and AgNPs released from the nanofibers [52]. One milliliter of the release medium was digested with 9 mL of 65% nitric acid using a microwave device.

Calibration was performed with external standards in the range of 0.5–100 ppb. Each measurement was performed in triplicate to ensure accuracy and reproducibility.

The in vitro release behavior of the Glucantime- and AgNPs-loaded tri-layer composite nanofiber was evaluated using different mathematical models commonly applied to describe drug release from polymeric delivery systems. The experimental release profiles, expressed as the cumulative fraction of drug released as a function of time, were fitted to zero-order, first-order, Higuchi, and Korsmeyer-Peppas kinetic models to investigate the release characteristics [53].

The zero-order kinetic model was applied to evaluate drug release occurring at a constant rate, independent of concentration, as described in Equation (2). The first-order kinetic model was used to describe concentration-dependent release behavior according to Equation (3). The Higuchi model was employed to investigate diffusion-controlled drug release from the nanofiber matrix based on Fickian diffusion, as expressed in Equation (4). To further elucidate the drug release mechanism from the nanofiber system, the initial portion of the release data ($M_{t}/M_{\infty }\leq 0.6$) was analyzed using the Korsmeyer-Peppas model, as shown in Equation (5):

The kinetic models used in this study are given as follows:

(2) $$Q_{t}=k_{0}.t$$

(3) $${lnQ}_{t}={lnQ}_{0}-k_{1}.t$$

(4) $$Q_{t}=k_{h}.t^{1/2}$$

(5) $$\frac{M_{t}}{M_{\infty }}=k_{p}.t^{n}$$

where $Q_{t}$ represents the percentage of drug released at time t, $Q_{0}$ is the initial drug concentration, and $k_{0}$, $k_{1}$, $k_{h}$, and $k_{p}$ are the corresponding release rate constants. The ratio $M_{t}/M_{\infty }$ denotes the fraction of drug released at time *t*, and n is the release exponent indicative of the drug release mechanism.

### 2.5. Biological Evaluation

#### 2.5.1. Antioxidant Activity

The reactive oxygen species (ROS) scavenging capacity of the nanofibers was evaluated by determining their antioxidant activity using the 2,2-diphenyl-1-picrylhydrazyl (DPPH) radical assay [54]. A 100 µM DPPH solution was freshly prepared in ethanol and used as the free radical source. For the assay, nanofiber samples were placed into separate centrifuge tubes, each containing 500 µL of distilled water and 500 µL of DPPH solution. A control sample was prepared by mixing 500 µL of DPPH solution with 500 µL of distilled water in the absence of nanofibers. All tubes were incubated at room temperature in the dark for 24 h to prevent light-induced degradation of the DPPH radicals [55]. After incubation, the solutions were analyzed spectrophotometrically at 517 nm using a UV–Vis spectrophotometer (Libra Biochrom, Cambridge, UK) to determine the decrease in absorbance. The antioxidant activity (%) of the nanofiber samples was calculated using the formula provided in Equation (6):

(6) $$DPPH\;scavenging\;rate\;(\%)\;=\;\frac{A_{c}-A_{s}}{A_{c}}\;\times \;100$$

where *A_c_* is the control absorbance and *A_s_* is the sample absorbance. The antioxidant activity study was performed in triplicate.

#### 2.5.2. Biocompatibility Evaluation

Preparation of test samples: During the MTT test, samples were prepared at various concentrations and added to the wells for analysis. An indirect method was used to ensure homogeneous distribution of the biomaterial obtained in nanofiber form. In the indirect method, nanofibers were incubated in 2 mL of cell line-appropriate medium for 24 h, and this release medium was applied to the cells during the experiment. AgNPs and Glucantime were diluted until they reached the predetermined concentrations. All samples were UV sterilized for 1 h before use. MTT tests were performed separately for 24 and 72 h. The nanofibers consisted of five different groups, and all experiments were performed on the following groups.

The experimental groups included PVA/Gel/PVA, PVA-AgNPs/Gel/PVA, PVA-AgNPs/Gel-Glu1.2%/PVA, PVA-AgNPs/Gel-Glu2.4%/PVA, PVA-AgNPs/Gel-Glu3.6%/PVA nanofibers, AgNPs at concentrations of 1, 10, 50, 100, 200, and 400 μg/mL, Glucantime at 50, 100, 200, and 400 μg/mL, and a control group.

Assessment of cell viability: L929 cells were cultured in DMEM-F12 medium supplemented with 10% FBS. Cells in the cryobank were thawed in a 37 °C water bath and transferred to fresh medium. The suspension was centrifuged at 1000 rpm for 5 min. After confirming that a pellet had formed at the bottom, the supernatant was removed. Five milliliters of DMEM-F12 medium were added to the pellet, and cells were seeded into T25 flasks. Cells were routinely monitored using an inverted microscope (CKX41, Olympus Corporation, Tokyo, Japan) and incubated under standard culture conditions (37 °C, 5% CO_2_, 95% humidity). When the flask reached 90% confluence, the medium was removed, and the cells were washed with PBS for passaging. One milliliter of trypsin solution was added, and the flask was incubated for 5 min. After microscopic confirmation that the cells had detached from the surface, 4 mL of medium was added to neutralize the trypsin. The cell suspension was transferred to a Falcon tube and centrifuged at 1000 rpm for 5 min. Cells were then counted using a Thoma slide for use in experiments. From the counted culture, 1 × 10^5^ cells per well were seeded into 96-well microplates in a final volume of 100 μL. After 24 h of incubation, the medium from each well was removed, test samples were added, and one well without a sample was designated as the control. The plates were incubated at 37 °C in a 5% CO_2_ atmosphere. At 24 and 72 h of incubation, 10 μL of sterile MTT solution (10 mg/mL) was added to each well, followed by a 4 h incubation under standard conditions. Upon microscopic confirmation of formazan crystal formation, 100 μL of DMSO was added to each well to dissolve the crystals, and the plates were incubated for another 30 min under the same conditions. Absorbance was measured at 570 nm using an ELISA reader (Thermo Scientific, Waltham, MA, USA). Cell viability in the wells was expressed as a percentage relative to the control group (considered 100%), and subsequent calculations and statistical analyses were performed on data obtained from three independent experiments [56,57].

#### 2.5.3. Antileishmanial Activity

Determination of antileishmanial activity in promastigote and amastigote culture: Antileishmanial activity assays were conducted at the Yıldız Technical University Cell Culture Laboratory using the CL strain of *Leishmania tropica* (MHOM/TR/99/EP39). Parasite cultures were maintained in RPMI 1640 medium supplemented with 10% FBS and incubated at 27 °C in a CO_2_-free incubator. Parasite growth and morphology were observed daily under an inverted microscope, and subcultures were performed weekly. Subsequently, 7.5 mL of medium was seeded into the flask, and parasites recovered from the culture were seeded onto fresh medium at a density of 3 × 10^5^ parasites/mL [58]. An indirect method was used to ensure homogeneous distribution of the nanofiber-based biomaterial. For this purpose, the nanofibers were incubated in 2 mL of FBS-free RPMI medium for 24 h to obtain the release medium. The experimental groups included PVA/Gel/PVA, PVA-AgNPs/Gel/PVA, PVA-AgNPs/Gel-Glu1.2%/PVA, PVA-AgNPs/Gel-Glu2.4%/PVA, PVA-AgNPs/Gel-Glu3.6%/PVA nanofibers, AgNPs at concentrations of 25, 50, 100, 200, and 400 μg/mL, Glucantime at 50, 100, 200, and 400 μg/mL, and a control group.

Determination of antileishmanial activity on *L. tropica* promastigotes: Antileishmanial activity testing on promastigotes was performed in Eppendorf tubes. Parasites and culture medium were transferred to a falcon tube to obtain a final density of 1 × 10^6^ parasites/mL. Then, 1 mL of medium containing 1 × 10^6^ parasites was added to each Eppendorf tube. Samples obtained by the indirect method, by releasing the nanofiber-based biomaterial for 24 h, were applied to the parasites along with 100 μL of AgNPs and Glucantime at predetermined concentrations. Samples were incubated for 24 h. To detect parasites before counting, 200 μL of 2% formaldehyde was added to the Eppendorf tube, followed by 50 μL of the sample. The number of viable parasites was then determined using a Thoma hemocytometer. Viability studies were conducted in triplicate and viability rates were calculated relative to the control group [59].

Determination of antileishmanial activity in amastigote macrophage cultures: J774 macrophage cells from the cryobank were thawed in a 37 °C water bath, transferred to medium, and then cultured in RPMI-1640 medium supplemented with 10% FBS. The supernatant was discarded by centrifugation at 1000 rpm for 5 min. 5 mL of RPMI medium was then added to the cell pellet, and the cells were seeded in a T25 flask. Cells were monitored regularly using an inverted microscope. When the flask reached approximately 90% confluence, cells were detached by gently tapping the flask. To establish a parasite-infected macrophage culture, macrophages were first counted and seeded in a 6-well plate at a density of 2.5 × 10^4^ cells per well and then incubated for 24 h under standard culture conditions (37 °C, 5% CO_2_, 95% humidity). Promastigote cultures were used to infect macrophages. The counted promastigotes were added to each macrophage-containing well at a density of 3 × 10^5^ parasites per well. After a 4 h incubation to allow infection, the medium was removed to remove extracellular parasites, and test samples were applied. After 96 h of incubation, amastigote-infected macrophages were fixed with methanol, then stained with Giemsa and examined microscopically. The studies were conducted in triplicate and infection index was calculated based on the count results.

### 2.6. Statistical Analysis

All experiments were carried out using three replicates (*n* = 3), and data are expressed as mean ± standard deviation. For datasets involving group comparisons, including swelling behavior and biological assays, statistical analysis was performed using one-way analysis of variance (ANOVA) followed by appropriate post hoc multiple comparison tests. A value of *p* < 0.05 was considered statistically significant.

## 3. Results and Discussion

### 3.1. Preparation and Physicochemical Properties of AgNPs

The synthesis of AgNPs under a silver nitrate to propolis extract ratio of 18:2 (*v*/*v*), pH 9.5, temperature of 80 °C, and reaction time of 2 h resulted in a stable colloidal suspension with uniform nanoparticle characteristics. These conditions were determined based on a series of optimization experiments, the detailed outcomes of which are provided in the Supplementary Materials (Figures S1–S3 and Tables S1–S3).

**Figure 1:**
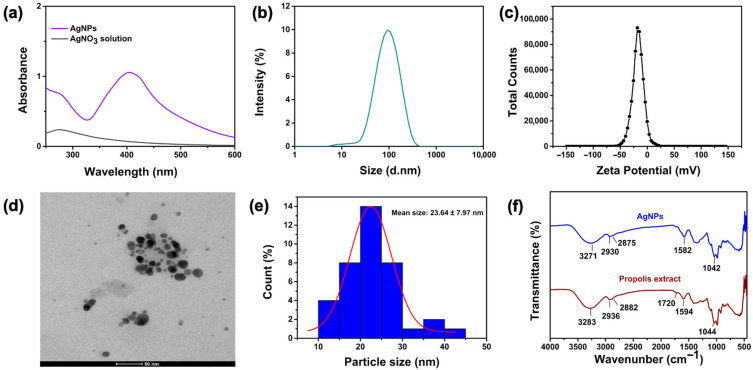
(**a**) UV–Vis spectrum of AgNPs and AgNO_3_, (**b**) DLS hydrodynamic size distribution of AgNPs, (**c**) zeta potential distribution of AgNPs, (**d**) TEM image of AgNPs, (**e**) particle size distribution of AgNPs. The histogram represents the particle size distribution, while the line represents the fitted distribution curve, and (**f**) FTIR spectra of the propolis extract and AgNPs.

UV–Vis spectroscopy confirmed the formation of AgNPs, as evidenced by a distinct surface plasmon resonance (SPR) peak at 405 nm (Figure 1a) [60]. This optical feature is characteristic of silver nanoparticles, whereas the AgNO_3_ precursor solution showed no notable absorption in the 200–800 nm range.

A PDI value lower than 0.3 is generally accepted as an indicator of a uniform particle size distribution, reflecting monodispersity in nanoparticles [61]. Dynamic light scattering (DLS) measurements (Figure 1b), indicated an average hydrodynamic diameter (Z-Ave) of 75.01 ± 0.453 nm with a polydispersity index (PDI) of 0.276 ± 0.005, suggesting a relatively narrow size distribution (PDI < 0.3). The zeta potential of the nanoparticles was determined to be −17.1 ± 0.693 mV (Figure 1c), reflecting a net negative surface charge and indicating acceptable colloidal stability in aqueous suspension. In colloidal systems, zeta potential values more negative than −30 mV or more positive than +30 mV are typically linked to strong electrostatic stabilization, while values ranging from ±15 to 30 mV reflect moderate stability arising from sufficient interparticle repulsion [62].

Transmission electron microscopy (TEM) analysis (Figure 1d) revealed predominantly spherical nanoparticles with an average particle size of 23.64 ± 7.97 nm (Figure 1e). The particle size observed by TEM was smaller than the hydrodynamic diameter measured by DLS, which is expected due to the absence of the hydration layer in TEM images [63].

FTIR spectroscopy in the infrared region (4000–400 cm^−1^) was employed to assess the capping/stabilizing role of the propolis extract (Figure 1f). FTIR analysis indicated that the propolis extract and the resulting AgNPs display largely overlapping infrared bands. The aqueous propolis extract displayed FTIR bands at 3283 cm^−1^ (broad O–H stretching of phenolic groups), 2936 and 2882 cm^−1^ (aliphatic C–H stretching of CH_2_/CH_3_), around 1700 cm^−1^ (C=O stretching vibration of carbonyl groups) [64], 1594 cm^−1^ (aromatic C=C vibrations), and 1044 cm^−1^ (C–O stretching of phenolics/alcohols) [65], consistent with FTIR profiles reported for propolis [35]. FTIR analysis showed that the AgNPs spectrum largely overlapped with that of the aqueous propolis extract. This spectral overlap is expected, as plant- and extract-mediated silver nanoparticles typically do not exhibit distinct Ag–O or Ag–Ag bands in the mid-infrared region due to the dominance of organic capping molecules [66]. Upon nanoparticle formation, small shifts and intensity changes were observed at 3271 cm^−1^ (O–H), 2930/2875 cm^−1^ (aliphatic C–H), and 1582/1042 cm^−1^ (aromatic/C=O), while the characteristic band around 1700 cm^−1^ (C=O stretching) disappeared in the AgNP spectrum. This disappearance suggests that carbonyl groups participated in the reduction in Ag^+^ ions and were consumed during nanoparticle synthesis. These spectral changes indicate that phenolic and carbonyl constituents bearing –OH, C=O, and C–O groups bind more strongly to the nanoparticle surface, acting not only as reducing agents but also as capping and stabilizing agents, thereby contributing to the colloidal stability of the synthesized AgNPs [67].

The use of a green synthesis strategy in this study was motivated by the incorporation of naturally derived biologically active reducing and stabilizing components into the nanoparticle system. Green synthesis routes, including propolis-mediated approaches, are known to play an effective role in shaping nanoparticle surface chemistry and influencing interactions with polymer matrices through the presence of bioactive organic constituents. Previous studies have shown that extract-mediated green synthesis can enhance the biological performance of AgNPs, likely due to synthesis-dependent surface characteristics [68,69]. Within this context, propolis-mediated AgNPs have the potential to contribute to the stability and biological response of the designed composite system.

### 3.2. Morphology and Elemental Composition of Composite Nanofibers (SEM–EDX)

**Figure 2:**
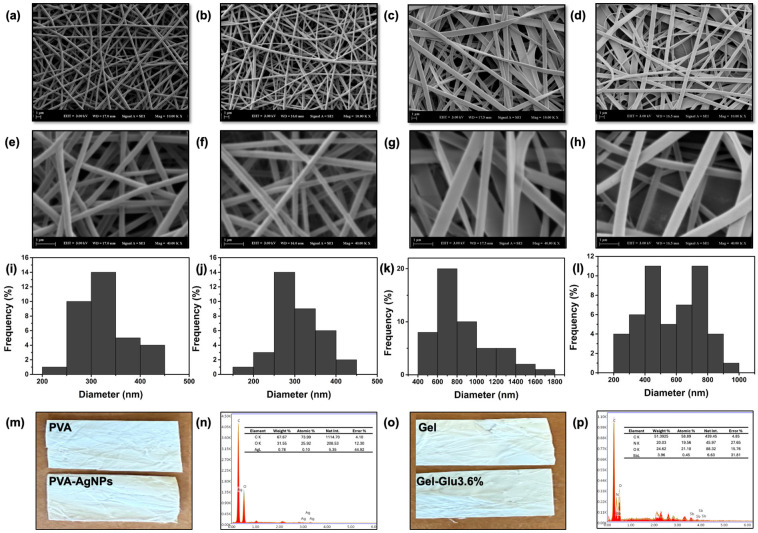
Morphological characterization of the nanofibers (**a**–**d**) SEM images of nanofibers PVA, PVA-AgNPs, Gel, and Gel-Glu3.6%, respectively, at a magnification of ×10.00, (**e**–**h**) SEM images of nanofibers PVA, PVA-AgNPs, Gel, and Gel-Glu3.6%, respectively, at a magnification of ×40.00, (**i**–**l**) Diameter distribution histograms of nanofibers PVA, PVA-AgNPs, Gel, and Gel-Glu3.6%, respectively, (**m**) Digital photographs of PVA, and PVA-AgNPs nanofibers, (**n**) EDX spectra of PVA-AgNPs, (**o**) Digital photographs of Gel, and Gel-Glu3.6% nanofibers, and (**p**) EDX spectra of Gel-Glu3.6%.

Homogeneous, bead-free PVA nanofibers were produced at 15 kV, a 15 cm tip collector distance, and 0.9 mL/h; the mean fiber diameter was 323 ± 53 nm, forming the inner layer of the multilayer wound dressing (Figure 2a,e). The corresponding fiber diameter distribution histogram is presented in Figure 2i. These diameters are consistent with the typical range reported for electrospun PVA nanofibers [70]. The uniform, bead-free, tubular and smooth PVA-AgNPs nanofibers were obtained at a flow rate of 0.6 mL/h, a distance of 15 cm, and an applied voltage of 17 kV (Figure 2b,f). The mean fiber diameter was 306 ± 55 nm, constituting the outermost layer of the multilayer wound dressing, with the corresponding diameter distribution histogram shown in Figure 2j. Adding AgNPs to the electrospinning solution yielded noticeably finer fibers without compromising the uniform, bead-free structure. As conductivity increases, the jet carries more positive charge, repulsive forces grow, the jet stretches further, and the resulting fibers are thinner [71,72]. The macroscopic appearance of the electrospun nanofiber mats was evaluated by digital photography, and Figure 2m shows digital photographs of the PVA and PVA-AgNPs nanofibers. The flat/ribbon and bead-free Gel nanofibers were produced at 18 kV, a 15 cm tip–collector distance, and 0.48 mL/h; the mean fiber diameter was 852 ± 290 nm (Figure 2c,g), which is consistent with values reported for electrospun gelatin nanofibers [40]. The corresponding fiber diameter distribution histogram is presented in Figure 2k.

In line with Topuz and Uyar [40], the ribbon-like morphology observed in our gelatin mats arises from the rapid evaporation of formic acid during electrospinning, which promotes surface skin formation and jet collapse, thereby yielding flat fibers under higher voltage and gelatin concentration conditions. The macroscopic appearance of the Gel nanofiber is shown in Figure 2o. Incorporation of Glucantime at 3.6% (*w*/*w*) into the Gel solution, followed by electrospinning under identical conditions, yielded fibers with a mean diameter of 578 ± 205 nm, forming the middle layer of the multilayer wound dressing (Figure 2d,h). Figure 2l shows the diameter distribution histogram of the Gel-Glu3.6% nanofiber, while Figure 2o presents its digital photograph. Introducing Glucantime led to a reduction in fiber diameter. This behavior matches that of ionic additives, where increased solution conductivity strengthens jet stretching and narrows the resulting fiber diameter [73]. The decrease in fiber diameter upon Glucantime addition can be attributed to its partially ionic nature [74], as meglumine antimonate partially dissociates into charged species in aqueous media, increasing the electrical conductivity of the spinning solution.

EDX spectra exhibited the characteristic Ag L-series lines (~3 keV), confirming the presence of silver within the PVA nanofibers (Figure 2n) [75]. EDX spectra acquired on the fibers exhibited the characteristic Sb L-series lines (~3.6–4.1 keV) [76], confirming the presence of antimony within the Gel mats (Figure 2p). Given that Sb originates from Glucantime in our formulation, these data support drug incorporation.

**Figure 3:**
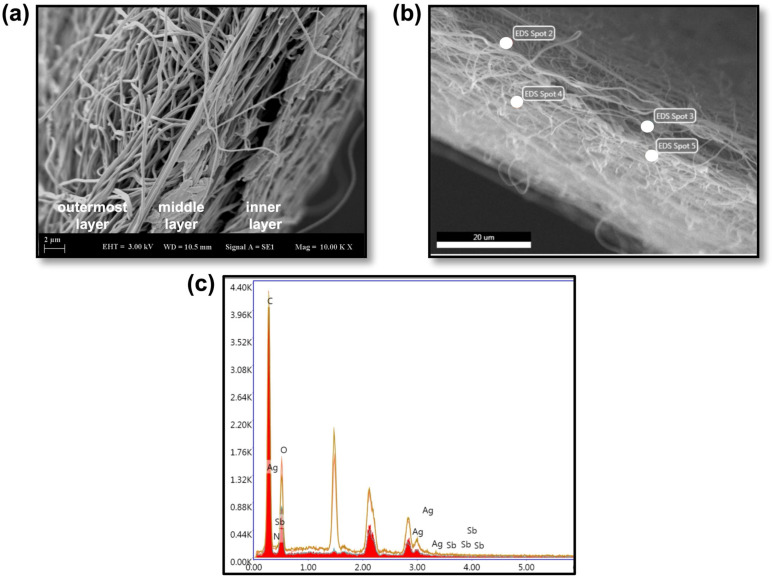
(**a**) SEM image of PVA-AgNPs/Gel-Glu3.6%/PVA nanofibers, (**b**,**c**) EDX analysis of PVA-AgNPs/Gel-Glu3.6%/PVA nanofibers.

As shown in Figure 3a, the tri-layer architecture of the composite nanofiber dressing is clearly resolved in the SEM micrograph, with well-defined interfaces between layers. EDX spectra exhibited the characteristic Ag L-series (~3.0 keV) and Sb L-series (~3.6–4.1 keV) lines [77], confirming the presence of silver and antimony within the electrospun nanofibers (Figure 3b,c). Given that antimony in our formulation originates solely from Glucantime, these data are consistent with drug incorporation.

### 3.3. FTIR Analysis

**Figure 4:**
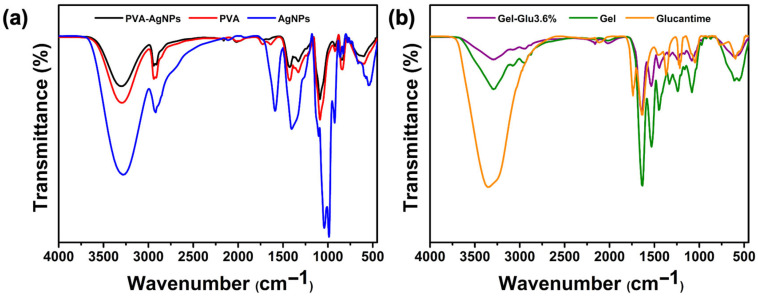
FTIR spectra of (**a**) AgNPs, PVA nanofibers and PVA-AgNPs nanofibers, (**b**) Glucantime, Gel nanofibers and Gel-Glu3.6% nanofibers.

The FTIR spectrum (Figure 4a) of neat PVA nanofibers exhibited a broad absorption band at 3299 cm^−1^, attributed to O–H stretching vibrations of hydroxyl groups involved in intermolecular hydrogen bonding. The peak at 2939 cm^−1^ corresponded to the stretching vibrations of alkyl C–H groups. A characteristic band at 1726 cm^−1^ indicated residual acetate groups from incomplete hydrolysis, while the absorption observed at 1641 cm^−1^ was assigned to H–O–H bending of adsorbed water, consistent with the hygroscopic nature of PVA. The bands at 1429 and 1331 cm^−1^ were related to CH_2_ bending and C–H deformation vibrations, respectively. A strong band at 1088 cm^−1^ was ascribed to C–O stretching of the polymer backbone. Additionally, the peaks observed at 920 and 837 cm^−1^ reflected the presence of unhydrolyzed vinyl acetate units within the PVA structure [48,78]. Overall, these results confirm the characteristic spectral features of PVA and indicate partial hydrolysis of the polymer. Upon incorporation of AgNPs, the intensity of the O–H stretching band decreases, while the broad O–H envelope in the 3000–3500 cm^−1^ region shows no pronounced shift in peak position. In addition, the band around 1088 cm^−1^ exhibits a decrease in relative intensity. These features indicate hydrogen-bond reorganization and interfacial interactions between PVA hydroxyls and AgNP surfaces/capping ligands, accompanied by a modest reduction in chain ordering. Metallic silver itself is IR-silent; the bands observed in the AgNPs trace arise from the organic capping/stabilizer, namely propolis. No new strong absorptions attributable to covalent bond formation are observed, supporting that the composite is governed by non-covalent polymer-nanoparticle interactions while retaining the chemical identity of PVA.

FTIR spectra (Figure 4b) of neat gelatin nanofibers show the expected protein bands: a broad amide-A envelope (N–H/O–H stretching, 3200–3500 cm^−1^) [79], CH_2_ stretching near ~2940 cm^−1^, a strong amide I band (1632.65 cm^−1^; C=O stretching), amide II (1530.78 cm^−1^; N–H bending/C–N stretching), and weaker amide III/C–O features between 1300 and 1000 cm^−1^ [80]. The Glucantime spectrum exhibits a broad absorption band in the 2800–3400 cm^−1^ region is attributed to the NH_2_^+^ stretching vibrations characteristic of the protonated amine groups in the drug structure. The absorption at 1631 cm^−1^ can be assigned to H–O–H bending and/or C–O stretching, while the strong band at 1047 cm^−1^ corresponds to overlapping C–O and Sb–O–C stretching vibrations of the meglumine antimoniate structure [81]. The FTIR spectra of Gel-Glu3.6% nanofiber confirm that the characteristic amide bands of gelatin (amide-A, amide I, and amide II) are preserved after the incorporation of Glucantime. Compared to pure gelatin, noticeable changes in band intensity are observed in the Glucantime-loaded nanofiber. In the Amide A region (3200–3500 cm^−1^), the Gel-Glu3.6% nanofiber exhibits more pronounced absorption than Gel nanofiber. This increase in absorption indicates that the –NH and –OH groups of gelatins participate in interactions with Glucantime, likely through hydrogen bonding, which enhances vibrational absorption in this region. A similar behavior is observed for the Amide I band located 1641.60 cm^−1^, which is assigned to C=O stretching vibrations. Upon Glucantime incorporation, this band shows stronger absorption intensity, while no noticeable shift in peak position or appearance of new absorption bands is observed. This indicates that the chemical backbone of gelatin remains intact and that Glucantime incorporation does not lead to the formation of new covalent bonds. In the 1200–1000 cm^−1^, the spectrum of Gel-Glu3.6% differs from that of pure gelatin and shows partial similarity to the spectrum of Glucantime. Although no new sharp absorption peaks are observed, changes in spectral pattern and band shape indicate contributions from Glucantime-related vibrational modes within the gelatin matrix. Due to the relatively low concentration of Glucantime compared to gelatin and the overlap of vibrational bands in this region, drug-specific signals remain limited [43]. FTIR analysis did not reveal the appearance of any additional strong absorption bands, suggesting that the chemical structure of gelatin was preserved and that its interaction with Glucantime occurs predominantly through non-covalent forces such as hydrogen bonding and electrostatic interactions.

### 3.4. Thermogravimetric Analysis

**Figure 5:**
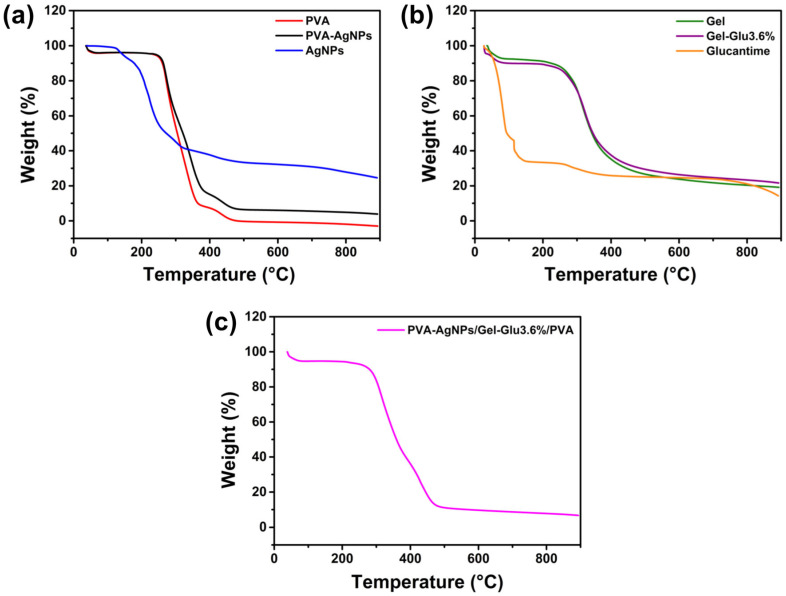
TGA thermogram of (**a**) AgNPs, PVA nanofibers and PVA-AgNPs nanofibers, (**b**) Glucantime, Gel nanofibers and Gel-Glu3.6% nanofibers, (**c**) PVA-AgNPs/Gel-Glu3.6%/PVA nanofibers.

Thermal degradation behavior of the produced materials was analyzed using TGA, and the corresponding results are displayed in Figure 5. The TGA and DTG thermal curves of all samples are provided in the Supplementary Materials (Figure S4). The associated thermal degradation parameters are summarized in Table 2.

To better interpret the effect of AgNPs on the polymer matrix, the thermal stability of the nanoparticles alone was also analyzed. The thermogravimetric analysis confirmed that the propolis-mediated AgNPs undergo a four-step decomposition process (Figure 5a). The first degradation stage occurred in the temperature range of 100–184 °C, and a mass loss of 11.39% was observed due to the removal of water from the structure. The second degradation stage occurred in the range of 184–280 °C, with a maximum degradation temperature of 210 °C, and a mass loss of 51.30% was detected in this stage. The third degradation stage occurred in the temperature range of 280–340 °C, with a maximum degradation temperature of 304 °C, and a mass loss of 59.40% was determined in this stage. The fourth degradation stage occurred in the range of 340–535 °C, with a maximum degradation temperature of 416 °C, and a total mass loss of 67.12% in this stage. These degradation stages are reported to be caused by the thermal degradation of corresponding to the degradation of phenolic compounds, flavonoids, and other organic constituents of propolis that served as reducing and stabilizing agents [82]. Heating the sample to 900 °C resulted in a residual mass of 24.61%. When only the TGA thermogram of propolis in the literature [83] is examined, it is observed that the structure undergoes approximately 67% degradation at around 400 °C. In contrast, the thermogram of AgNPs synthesized using propolis shows a similar level of mass loss at approximately 535 °C. This temperature difference is thought to be due to the strong interactions between Ag ions and the organic compounds in the propolis structure [84]. When comparing propolis and AgNPs synthesized via propolis, a mass difference of approximately 4% was determined at 900 °C. This difference is considered to be due to the silver ions present in the structure.

According to the TGA thermogram, PVA nanofibers exhibited a four-stage degradation profile. The first stage, occurring between 25 and 56 °C, corresponded to the removal of moisture and volatile compounds, resulting in 4.54% weight loss. Beyond this temperature, no considerable change was observed until 230 °C. The second stage, within 231–298 °C, was attributed to the dehydration of hydroxyl groups and acetic acid elimination, as also reported in the literature [85] with a maximum degradation temperature of 274 °C, and a mass loss of 45.75%. The third stage, observed between 299 and 393 °C with a maximum degradation temperature of 335 °C corresponded to the scission of the main polymer chain, leading to a weight loss 92.29%. The final stage, occurring within 393–479 °C, with a maximum degradation temperature of 433 °C was associated with the degradation of carbonaceous residues, after which the weight of PVA nanofibers nearly reached zero.

When the thermograms of PVA and PVA-AgNPs nanofibers are compared, a comparable degradation profile is exhibited. In the first degradation step, corresponding to the removal of water, a mass loss of 4.54% was observed in AgNPs-containing PVA nanofibers, similar to that observed for pristine PVA nanofibers. However, from the second degradation step onwards, a significant decrease in the amount of mass loss occurred in AgNP-containing nanofibers. In this context, the mass loss decreased from 45.75% to 34.96% in the second step, from 92.29% to 84.84% in the third step, and from 99.97% to 93.68% in the fourth step. Upon heating to 900 °C, pristine PVA nanofibers were completely thermally degraded, whereas AgNP-containing PVA nanofibers retained approximately 3.86% residual mass. In the final degradation stage, increases were observed in the maximum degradation temperature (from 433 °C to 439 °C) as well as in the terminal degradation temperature (from 479 °C to 511 °C). Considering the AgNPs added to the mixture during the formation of PVA-AgNPs nanofibers, theoretical calculations based on AgNPs and PVA thermograms predicted that approximately 0.47% of the mass would remain. However, experimental results showed that 3.86% of the mass remained. Although these results may seem independent at first glance, they indicate that the incorporation of AgNPs into PVA nanofibers restricts polymer chain mobility, delays complete thermal decomposition, and thus improves the overall thermal stability of the system.

To better interpret the effect of Glucantime on the polymer matrix, the thermal behavior of the drug alone was also analyzed. For this purpose, since it could not be obtained in dry form, TGA of Glucantime was performed in liquid form. According to the results obtained, it was determined that the water in the structure was completely removed at 115 °C, and a mass loss of 67% occurred at this stage. Following the initial mass loss due to water removal, a second degradation was observed due to the decomposition of organic components in the structure, and the total mass loss at this stage was determined to be 75%. The residual mass observed at 900 °C was determined to be approximately 15%. The residual mass observed at elevated temperatures is most likely associated with inorganic structures, which may be related to the presence of antimony oxides in the composition [86].

The TGA thermogram of gelatin nanofiber showed a two-step degradation behavior (Figure 5b). The first stage occurred between 25 °C and 85 °C with a weight loss of about 10.30%, which can be attributed to the removal of moisture and volatile compounds. Above this temperature, no significant change was observed up to 230 °C. The second stage took place in the range of 230–606 °C and corresponded to the thermal degradation of the gelatin polymer chains. The maximum degradation temperature was recorded at 309 °C, and the total mass loss at this stage was determined to be 76.33%. When the sample was heated to 900 °C, it was determined that 80.83% of the total mass underwent thermal decomposition. These findings are consistent with literature reports, confirming the typical degradation mechanism of gelatin fibers, where initial water loss is followed by the decomposition of protein chains [87,88,89].

The TGA thermograms of Gel-Glu3.6% nanofibers containing Glucantime were found to be highly similar to those of Gel nanofibers (Figure 5b). Mass loss of 9.90% occurred in the 25–92 °C range, and 73.65% in the 187–603 °C range, with a maximum decomposition temperature of 325 °C. After heating to 900 °C, 78.35% of the structure was undergone thermal decomposed. It was determined that the addition of Glucantime to the nanofiber structure resulted in approximately a 2.5% reduction in total mass loss. However, it was found that the maximum degradation temperature observed in the second degradation step increased by approximately 16 °C. This reduction in mass loss is thought to be due to the fact that the antimony element in the Glucantime structure remains stable at high temperatures without undergoing thermal decomposition in its oxide form. Theoretically, considering the amounts of gelatin and added Glucantime, and based on the thermal decomposition data of Gel nanofibers and Glucantime alone, calculations predict an approximate residual mass of 18.99%. The experimentally obtained result is a residual mass of 21.65%. A good agreement is observed between theoretical and experimental results. The observed limited mass difference is thought to be due to the interaction of the degradation products formed during the thermal decomposition processes of gelatin and Glucantime.

The TGA thermogram of PVA-AgNPs/Gel-Glu3.6%/PVA nanofibers is presented in Figure 5c. The layered fibers exhibited a three-step degradation profile. The first stage, which took place between 25 and 76 °C, corresponded to the removal of moisture and volatile compounds, resulting in a mass loss of 6.23%. Water loss was found to be exactly between PVA-AgNPs and Gel-Glu3.6% nanofibers, confirming the multilayer structure. Above this temperature, no significant mass loss was observed up to 225 °C. Between 225 and 391 °C, there was a second degradation step occurring at a maximum degradation temperature of 317 °C, resulting in a mass loss of 61.32%. This step occurred over a much wider temperature range and with much greater mass loss than in PVA-AgNPs nanofiber. It is thought that this may be due to the fact that Gel nanofibers undergo degradation at a higher rate over the same temperature range. The maximum degradation temperature was determined to be quite close to that of Gel nanofibers. The third degradation step, observed in the temperature range of 391–536 °C, reached its maximum degradation temperature at 408 °C, resulting in 89.58% mass loss at this stage. A large portion of this mass loss is due to the degradation of the PVA chains within the nanofibers. As a result of the heating process carried out up to 900 °C, it was determined that 6.62% of the structure remained as residue. This value is higher than that of PVA-AgNP nanofibers but lower than that of Gel-Glu3.6% nanofibers. This behavior is attributed to the three-layered architecture of the structure, in which two layers are composed of PVA nanofibers, while only one layer consists of Gel nanofibers.

Taken together, the TGA results provide a clear understanding of the thermal behavior of the tri-layer wound dressing across the investigated temperature range. The absence of pronounced mass changes at low temperatures, including physiological conditions, together with the well-defined degradation behavior at higher temperatures, highlights the relevance of the thermal profile for storage, handling, and processing considerations in biomedical applications.

**Table 2:** TGA results of the samples. S1: AgNPs; S2: Propolis; S3: PVA nanofiber; S4: PVA-AgNPs nanofiber; S5: Glucantime; S6: Gel nanofiber; S7: Gel-Glu3.6% nanofiber; S8: PVA-AgNPs/Gel-Glu3.6%/PVA nanofiber.

Sample	First Stage			Second Stage			Third Stage			Fourth Stage			Residue at 900 °C (%)
	Range (°C)	Peak (°C)	Weight Loss (%)	Range (°C)	Peak (°C)	Weight Loss (%)	Range (°C)	Peak (°C)	Weight Loss (%)	Range (°C)	Peak (°C)	Weight Loss (%)	
S1	100–184	130	11.39	184–280	210	51.30	280–340	304	59.40	340–535	416	67.12	24.61
S2	25–140		2.4	239–395	348	67.70							20.51 [83]
S3	25–65		4.54	231–299	274	45.75	299–393	335	92.29	393–479	433	99.97	0
S4	25–90		4.58	231–293	270	34.96	293–399	339	84.84	399–511	439	93.68	3.86
S5	25–114	82	54.08	114–173	115		250–510	275	74.96				14.26
S6	25–85		10.30	230–606	309	76.33							19.17
S7	25–92	56	9.90	187–603	325	73.65							21.65
S8	25–76		6.23	225–391	317	61.32	391–536	408	89.58				6.72

### 3.5. Contact Angle Analysis

The contact angle (CA) is a quantitative measure of surface hydrophilicity. Contact angle quantifies wettability; in nanofiber studies, surfaces are typically classified as superhydrophilic (CA < 10°), hydrophilic (10–90°), hydrophobic (90–150°), and superhydrophobic (>150°) [90].

**Figure 6:**
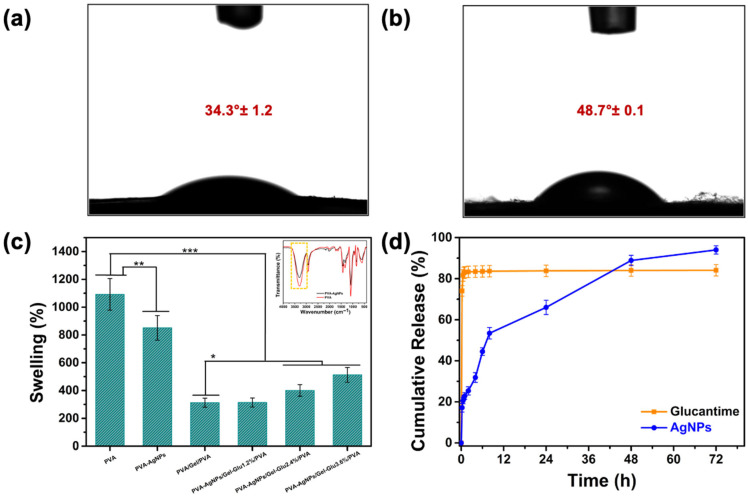
(**a**) Contact angle of PVA nanofibers, (**b**) contact angle of PVA-AgNPs nanofibers, (**c**) swelling ratio (%) of nanofibers, and (**d**) release kinetics of Glucantime and AgNPs from nanofibers. FTIR spectra of PVA nanofibers and PVA-AgNPs nanofibers were included in panel (**c**) to facilitate discussion. Release and swelling measurements are reported as mean ± standard deviation (SD) based on three independent experiments (*n* = 3). * *p* < 0.05, ** *p* < 0.01, and *** *p* < 0.001.

Contact angle analysis revealed that the contact angles of PVA nanofibers (Figure 6a), and PVA-AgNP nanofibers (Figure 6b) were 34.3° ± 1.2 and 48.7° ± 0.1 degrees, respectively. This behavior can be explained by surface chemistry: the high density of surface-accessible hydroxyl (–OH) groups originating from the PVA backbone promotes strong hydrogen bonding with water molecules, accounting for the inherently hydrophilic nature of pristine PVA nanofiber surfaces. Ag nanoparticles interact with PVA hydroxyl groups, effectively occupying a portion of the polar –OH sites at the fiber–air interface. In addition, their organic capping layers tend to enrich the surface with less-polar moieties, which lowers the polar component of the surface free energy and leads to a higher water contact angle [91].

In the current multilayer wound dressing design, only the pure PVA nanofiber layer is in direct contact with the wound surface. This layer retains a relatively low contact angle (34.3° ± 1.2), indicating a hydrophilic character favorable for the wound interface. Such hydrophilicity supports moisture retention at the wound site and promotes fluid absorption, both of which are key factors in the wound healing process. Meanwhile, the PVA-AgNPs layer, with a slightly higher contact angle (48.7° ± 0.1) and thus reduced hydrophilicity compared to neat PVA nanofibers, is positioned away from the wound bed. Within this multilayer structure, the PVA-AgNPs layer provides antileishmanial activity and functions as a physical barrier. Therefore, the distinct wettability profiles of the two layers are complementary: the hydrophilic PVA layer ensures a moist and biocompatible wound environment, while the relatively less hydrophilic PVA-AgNPs layer contributes protective and therapeutic functionality. The different wettability profiles of the layers provide complementary functions within the multilayer architecture; the hydrophilic PVA layer optimizes interaction with the wound, while the PVA-AgNPs layer with lower hydrophilicity provides protective and therapeutic functionality.

### 3.6. Water Absorption Capacity

Effective wound care requires removal of excess exudate without disrupting a moist environment. Accordingly, the water-uptake (WU) capacities of our electrospun materials were quantified (Figure 6c). Neat PVA nanofibers showed a WU of 1092.26 ± 80.43%, which decreased to 850.97 ± 75.37% upon AgNPs incorporation. We therefore quantified the swelling of the electrospun mats as surrogate metrics of exudate management. Incorporating silver nanoparticles into the PVA-based electrospun matrix significantly reduced the equilibrium water uptake compared with neat PVA (*p* < 0.01), consistent with prior reports on Ag-loaded electrospun systems [92].

This effect is ascribed to interfacial coordination/hydrogen-bonding that sequesters a fraction of accessible –OH sites together with a filler-like, physical-junction effect that decreases free volume and increases transport tortuosity collectively limiting solvent ingress and osmotic swelling [93]. This interpretation is further supported by the FTIR spectra, in which the –OH stretching band is clearly observed in neat PVA but appears with reduced intensity in PVA-AgNPs nanofibers, indicating that the incorporation of AgNPs reduces the number of accessible hydroxyl groups. The water uptake ability of the PVA/Gel/PVA nanofibers was 312.90 ± 32.50%. With increasing Glucantime content in the gelatin core, the water uptake capacity of the tri-layer nanofiber constructs showed a marked increase, reaching 313.92 ± 32.60% for PVA-AgNPs/Gel-Glu1.2%/PVA, 400.77 ± 41.62% for PVA-AgNPs/Gel-Glu2.4%/PVA, and 512.75 ± 53.25% for PVA-AgNPs/Gel-Glu3.6%/PVA nanofibers. However, the differences between group PVA/Gel/PVA and group PVA-AgNPs/Gel-Glu1.2%/PVA were not statistically significant (*p* > 0.05).

Incorporation of AgNPs lowers swelling in PVA layers; however, the tri-layer PVA-AgNPs/Gel-Glu3.6%/PVA construct showed higher total water uptake than the PVA/Gel/PVA control (*p* < 0.05). The pronounced swelling of the drug-loaded system can be attributed not only to the partially ionic and hydrophilic nature of Glucantime, which creates a strong osmotic gradient for water uptake, but also to morphological changes induced by drug incorporation. Specifically, the addition of Glucantime reduced fiber diameter, thereby increasing the porosity and interconnectivity of the gelatin core. This synergistic effect of enhanced osmotic driving force and improved network permeability facilitated greater fluid imbibition, resulting in higher swelling than the PVA/Gel/PVA, despite the structural stabilization imparted by AgNPs in the outer PVA layers.

In the multilayer wound dressing design, the neat PVA nanofiber layer in direct contact with the wound surface supports rapid fluid uptake and moisture retention due to its highly hydrophilic nature. The intermediate gelatin layer containing Glucantime exhibits a controlled swelling behavior, which can be attributed to the presence of amino (–NH_2_) and carboxyl (–COOH) functional groups capable of forming hydrogen bonds and electrostatic interactions with water molecules. This hydrated gelatin network provides a suitable microenvironment for drug diffusion and release. In contrast, the outer PVA-AgNPs layer, which exhibits lower hydrophilicity and a more compact structure compared to the neat PVA nanofiber layer, functions as a physical barrier and contributes to maintaining the overall mechanical integrity of the dressing. Consequently, this layered architecture enables effective fluid absorption and swelling while preventing excessive expansion or uncontrolled dissolution of the nanofiber matrix.

### 3.7. In Vitro Release Profiles of Glucantime and Silver Nanoparticles

Before release study, the silver and Glucantime content of the PVA-AgNPs/Gel-Glu3.6%/PVA nanofibers were determined via ICP-MS. It was found that 10 mg of the nanofiber sample contained 2.53 μg of silver and 292.2 μg of Glucantime.

The release behavior of Glucantime and AgNPs from the PVA-AgNPs/Gel-Glu3.6%/PVA nanofibers was examined in phosphate-buffered saline (pH 7.4) at 37 °C to simulate physiological conditions. Figure 6d presents the release behavior of nanofibers loaded with Glucantime and AgNPs. Within the 72 h experimental period, the nanofibers released approximately 84% of Glucantime and 94% of AgNPs.

The Glucantime-loaded nanofibers exhibited a pronounced burst release, with approximately 74% released within the first 15 min, reaching 82% at 30 min. Beyond this point, the release profile plateaued and remained nearly constant up to 72 h. This rapid initial release can be attributed to the hydrophilic nature of Glucantime, which facilitates its diffusion through the nanofibrous matrix; similar burst phenomena for hydrophilic molecules have been reported in previous studies [94,95]. SEM analysis further supports this behavior, as Glucantime-loaded nanofibers displayed larger fiber diameters, which promote faster solvent penetration and drug diffusion. From a therapeutic standpoint, the initial burst release of Glucantime can be interpreted as a local loading dose that rapidly saturates the infected tissue and initiates prompt antiparasitic action. In cutaneous leishmaniasis, the parasite burden at the lesion site is typically high at the early stages of infection; therefore, rapid drug release ensures that Glucantime quickly reaches therapeutically effective concentrations, including the minimum inhibitory concentration (MIC), at the target site. This rapid achievement of therapeutic levels facilitates early reduction in actively proliferating *Leishmania* parasites and supports immediate treatment onset. Moreover, effective management of cutaneous leishmaniasis requires that antileishmanial agents penetrate infected macrophages and reach intracellular amastigotes located within parasitophorous vacuoles, necessitating sufficiently high local drug concentrations to overcome multiple biological barriers, including the macrophage membrane and intracellular compartmentalization [96]. Nevertheless, from a practical perspective, the pronounced initial release may necessitate more frequent dressing replacement during the early phase of treatment to maintain optimal drug levels, which should be considered in clinical applications.

In contrast, the AgNPs containing nanofibers showed a more gradual release behavior, with only 17% released at 15 min and a sustained increase over time, indicative of a controlled release pattern. The smaller diameters observed for AgNP-loaded nanofibers are consistent with restricted diffusion pathways, contributing to the slower and more sustained release. Upon hydration, the gelatin layer partially dissolved, whereas the PVA layer only swelled without dissolving, which contributed to the dual-phase release behavior of the multilayer structure. Together, these distinct release kinetics suggest that the combined system may provide both an early therapeutic effect from Glucantime and prolonged antileishmanial activity from AgNPs.

These findings can be directly attributed to the rational design of the tri-layer nanofiber architecture, in which each layer is assigned a specific functional role in controlling release behavior. The inner neat PVA layer, which is in direct contact with the lesion, serves as a moist interface that supports hydration of the wound surface and promotes close contact between the dressing and the affected tissue. The intermediate gelatin layer containing Glucantime readily hydrates due to its hydrophilic nature and functional groups, facilitating water uptake and drug diffusion. This characteristic accounts for the rapid initial release of Glucantime observed in vitro and is considered relevant for CL lesions, which often present with a high local parasite burden and may benefit from early localized drug availability. The outer PVA layer incorporating AgNPs provides an additional therapeutic function by contributing antileishmanial activity while also acting as a physical barrier. Compared to the gelatin layer, the PVA matrix is associated with a slower diffusion environment, resulting in a more gradual release profile of AgNPs. Furthermore, the presence of PVA layers on both sides of the gelatin core helps regulate swelling behavior and reduces premature dissolution of the drug-loaded layer, thereby maintaining the overall structural coherence of the fibrous dressing.

Following the evaluation of release profiles, the obtained experimental data were analyzed using mathematical release kinetics models to elucidate the release mechanism in more detail as shown in Table 3.

It was not possible to apply the Korsmeyer-Peppas model to Glucantime. The main reason for this is that the Korsmeyer–Peppas equation is only valid for the first 60% release region [97], and Glucantime quickly exceeds this limit by exhibiting a distinct burst release behavior. This rapid initial release resulted in the Glucantime release data not showing a significant agreement with the examined mathematical release kinetics models, thus preventing the Glucantime release from being adequately described by any kinetic model.

In contrast, it was determined that the release behavior of AgNPs could be described using mathematical release kinetics models. The AgNPs release data showed a particularly good fit to first order and Korsmeyer-Peppas models, as confirmed by the high R^2^ values obtained for these models. These findings demonstrate that the AgNPs release rate varies depending on the amount of AgNPs remaining in the system, and that the release behavior can be described using appropriate kinetic models.

The fact that the calculated *n* value within the scope of the Korsmeyer-Peppas model is below 0.5 reveals that the AgNPs release mechanism occurs predominantly via Fickian diffusion [94]. This result shows that AgNPs are released from the nanofiber matrix primarily through a diffusion-controlled mechanism, and that the structural properties of the polymer matrix play a decisive role in the release behavior.

### 3.8. Biological Evaluation

#### 3.8.1. Antioxidant Activity

**Figure 7:**
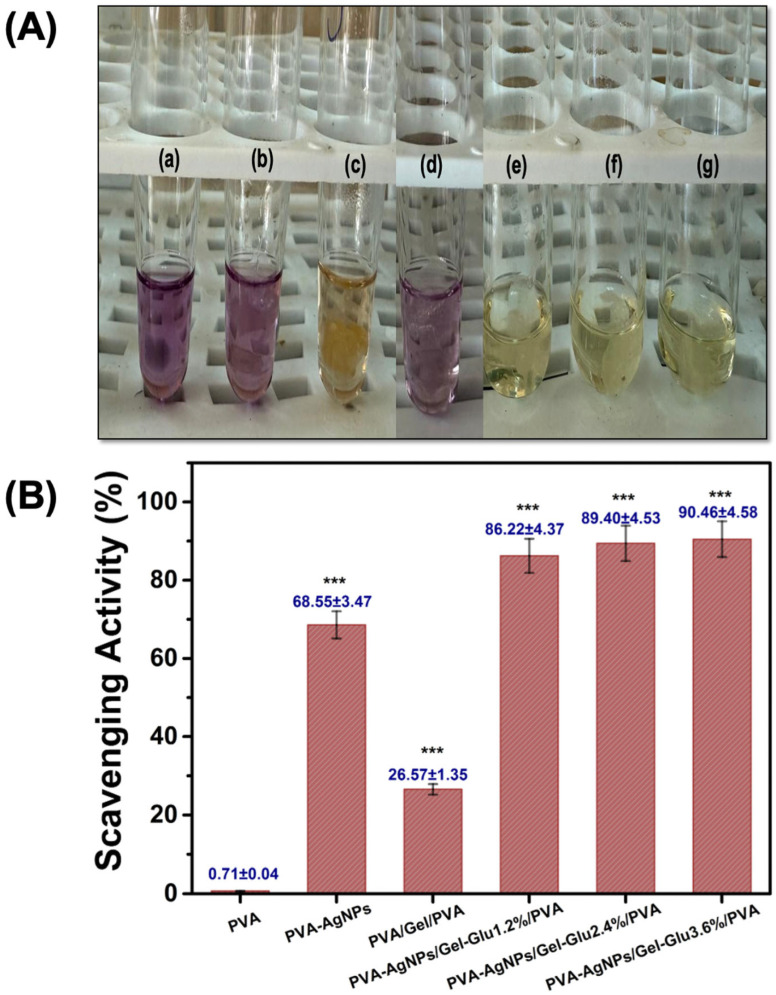
(**A**) DPPH decolorization for (a) control, (b) PVA, (c) PVA-AgNPs, (d) PVA/Gel/PVA, (e) PVA-AgNPs/Gel-Glu1.2%/PVA, (f) PVA-AgNPs/Gel-Glu2.4%/PVA, and (g) PVA-AgNPs/Gel-Glu3.6%/PVA nanofibers; and (**B**) DPPH radical scavenging activity (%) of the nanofibers. Data are reported as mean ± standard deviation (SD) obtained from three independent experiments (*n* = 3). *** *p* < 0.001 vs. PVA nanofiber.

Excess reactive oxygen species hinder healing; accordingly, we quantified the radical scavenging capacity of the electrospun nanofibers, with results summarized in Figure 7. While ROS/NO contribute to parasite killing, excessive oxidative stress in CL lesions impairs keratinocyte function and re-epithelialization [98]. Thus, incorporating moderate antioxidant capacity can protect host tissue and support closure without undermining therapy; notably, several antioxidants also display direct antileishmanial activity [99]. This relationship has been demonstrated in *Quercus infectoria* Olivier extract, which exhibits potent radical-scavenging capacity together with significant activity against *Leishmania major*, supporting the relevance of antioxidant-based approaches in cutaneous leishmaniasis management [100].

The DPPH clearances of PVA, PVA-AgNPs, PVA/Gel/PVA, PVA-AgNPs/Gel-Glu1.2%/PVA, PVA-AgNPs/Gel-Glu2.4%/PVA and PVA-AgNPs/Gel-Glu3.6%/PVA nanofibers were 0.71 ± 0.04, 68.55 ± 3.47, 26.57 ± 1.35, 86.22 ± 4.37, 89.40 ± 4.53 and 90.46 ± 4.58%, respectively. Incorporation of AgNPs increased the antioxidant (radical scavenging) activity of the PVA nanofiber mats (*p* < 0.001). The increase in antioxidant activity measured upon addition of AgNPs is attributed to the ability of nanoparticle surfaces and coating types to transfer electrons to free radical such as DPPH; this increase has been experimentally demonstrated in AgNPs-loaded membranes and green synthesized AgNPs [101,102]. In addition, nanofibers containing gelatin increased the antioxidant activity was probably due to free radical-scavenging peptide sequence present in gelatin (His-Gly-Pro-Leu-Gly-Pro-Leu) [103]. The enhanced radical-scavenging capacity of the composite formulations may therefore contribute to mitigating excessive oxidative stress in CL lesions, complementing their antileishmanial functionality.

#### 3.8.2. Biocompatibility Evaluation

**Figure 8:**
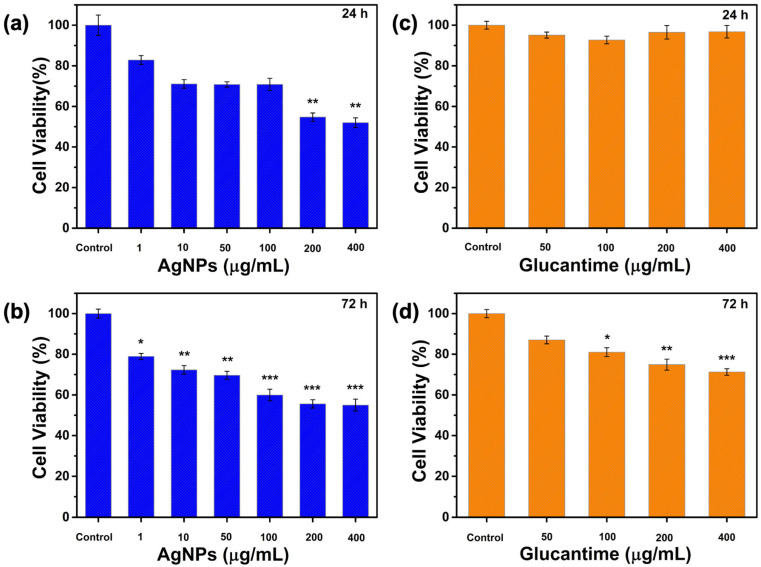
24 h and 72 h MTT assay results for different concentrations of AgNPs and Glucantime in L929 fibroblast cells. (**a**) 24 h MTT analysis of AgNPs, (**b**) 72 h MTT analysis of AgNPs, (**c**) 24 h MTT analysis of Glucantime, and (**d**) 72 h MTT analysis of Glucantime. The data are presented as mean ± standard deviation (SD) derived from three independent experiments (*n* = 3). * *p* < 0.05, ** *p* < 0.01, *** *p* < 0.001 vs. control.

**Figure 9:**
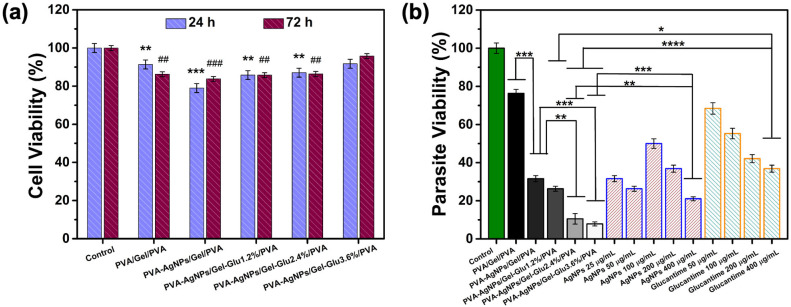
(**a**) 24 h and 72 h MTT assay results for L929 cells treated with nanofiber formulations. ** *p* < 0.01, and *** *p* < 0.001 compared with the control; ^##^ *p* < 0.01, and ^###^ *p* < 0.001 compared with the control, and (**b**) viability of *L. tropica* promastigotes after exposure to nanofibers with different compositions and to the sole application of AgNPs and Glucantime. Results are expressed as mean ± standard deviation (SD) based on three independent experiments (*n* = 3; * *p* < 0.05, ** *p* < 0.01, *** *p* < 0.001, and **** *p* < 0.0001).

The biocompatibility of the prepared nanofibers and the active ingredients loaded into the nanofiber was examined in the L929 fibroblast cell line by MTT analysis. MTT analysis for in vitro studies is a valuable analysis in terms of proving the existence of metabolic activity of cells and demonstrating cell viability. The amount of reduction in tetrazolium salt to purple formazan crystals by the mitochondria of cells that carry out their vital activities is parallel to the number of living cells. However, to measure the absorbance, the formazan crystals must be converted into a form suitable for measurement by applying DMSO. These processes were applied on the fibroblast cell line. The data obtained for cell viability in the absorbance measurements made at the end of the 24th and 72nd hours are shown in Figure 8 and Figure 9. 

When the results of the biocompatibility test and the one-way ANOVA results were examined, although cell viability rates decreased with increasing concentrations of AgNPs and Glucantime, high biocompatibility was found in nanofibers containing different concentrations of Glucantime versus a constant amount of AgNPs (*p* < 0.001). It was detected that none of the investigated nanofibers exhibited considerable toxicity over L929 fibroblast cells. The lower cytotoxicity observed for the AgNP-loaded fibers compared with free AgNPs suggests that incorporation of AgNPs within the polymer matrix limits direct nanoparticle–cell interactions, thereby reducing immediate cellular exposure to silver ions released from AgNPs. This behavior is consistent with previous studies reporting that polymer-embedded AgNP systems exhibit restricted nanoparticle mobility and more controlled silver ion release, which are associated with improved cytocompatibility in biomedical and wound-related applications [104,105].

The results show that various combinations of experimental groups do not have cytotoxic effects on the L929 cell line. Similar results were obtained in terms of cell viability and biocompatibility at 24 and 72 h. While the groups treated only with Glucantime showed higher viability rates (*p* < 0.001) than the AgNPs groups, nanofibers incorporating a constant amount of AgNPs and varying concentrations of Glucantime showed an increase in cell viability and a decrease in toxicity (*p* < 0.01). In the light of these results, it can be stated that the designed nanofiber biomaterial has high biocompatibility.

#### 3.8.3. Antileishmanial Activity

Determination of antileishmanial activity on *L. tropica* promastigotes: To study the antiparasitic effects of Glucantime and AgNPs-loaded nanofibers on *L. tropica* promastigotes, we analyzed them comparatively with promastigotes treated with different concentrations of AgNPs or different concentrations of Glucantime alone.

In this assay, the experimental groups were designed to explore potential combined effects included PVA-AgNPs/Gel/PVA (includes AgNPs, without Glu) and PVA-AgNPs/Gel-Glu1.2%/PVA, PVA-AgNPs/Gel-Glu2.4%/PVA, and PVA-AgNPs/Gel-Glu3.6%/PVA allowing comparison of systems in the presence and absence of Glucantime.

Promastigotes fixed in formaldehyde were counted, and parasite viability rates were calculated according to the control group (Figure 9b). According to the one-way ANOVA test, statistically significant differences were found in terms of promastigote viability between different formulations and all experimental groups (*p* < 0.001). This result shows that formulation change has a significant effect on parasite viability. According to the post hoc multiple comparison analysis, different concentrations of all tested nanofiber formulations, AgNPs, and Glucantime statistically significantly reduced parasite viability compared to the control group (*p* < 0.001). Comparisons between tri-layer nanofiber-based formulations with medium and high Glucantime concentrations (PVA-AgNPs/Gel-Glu2.4%/PVA and PVA-AgNPs/Gel-Glu3.6%/PVA) and systems containing free Glucantime (Glucantime 50–400 μg/mL) and free AgNPs (AgNPs 25–400 μg/mL) show that parasite viability is relatively lower in nanofiber-based formulations. With the increase in the concentration of Glucantime loaded into the nanofibers, notable decreases in the viability value of promastigotes were observed, especially at concentrations of 2.4 and 3.6% (*w*/*w*). However, the difference between these two groups was not statistically significant (*p* = 0.061). According to the calculations, these two formulations resulted in the same rate of decrease in promastigote cell viability, and a 10-folds reduction was established compared to the control group. Here, the formulation containing only PVA and Gel nanofibers had less effect on parasite viability compared to formulations containing AgNPs, which was interpreted as an indication of the fatal effect of AgNPs on promastigotes. On the other hand, it can be concluded that the combination of silver nanoparticle and Glucantime has a high antiparasitic effect on parasites and creates a synergistic effect. The dual-loaded tri-layer formulations (PVA-AgNPs/Gel-Glu2.4%/PVA and PVA-AgNPs/Gel-Glu3.6%/PVA) exhibited higher antileishmanial activity than the solely AgNPs-containing tri-layer fibers (PVA-AgNPs/Gel/PVA) under identical conditions. These two nanofibers were the most effective treatment formulations among test groups and they almost completely inhibited promastigote survival. This result has been attributed to the synergistic effect created by the addition of Glucantime to the fibers. However, this synergistic effect is interpreted based on comparative in vitro outcomes rather than a formal quantitative synergy analysis. When examining the possible mechanism of this synergistic effect, AgNPs have a high surface area-to-volume ratio and can also damage membrane structures containing sulfur and phosphorus through the released silver ions. Consequently, Glucantime whose active component is meglumine antimoniate can traverse the compromised membrane more readily, gaining access to the cytoplasm, where it suppresses enzymatic activity in the parasite. Furthermore, it may also induce damage to DNA and RNA, thereby reinforcing its inhibitory action. Although silver nanoparticles have been shown to have an inhibitory effect on parasites when applied alone [106], the risk of cytotoxicity to healthy cells due to metal ions remains a concern. The synergistic effect of AgNPs with low-dose Glucantime in a nanofiber structure suggests that the nanofiber system may be a platform that can contribute to safe and rapid healing in cutaneous leishmaniasis-related wounds.

**Figure 10:**
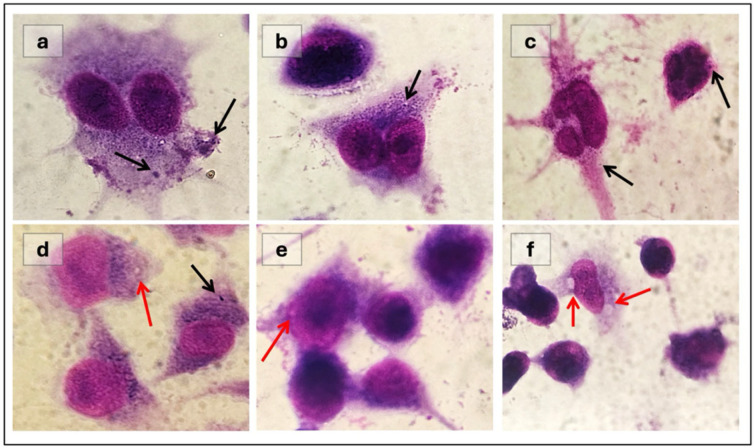
In vitro activity against intracellular amastigotes in macrophages. Photomicrographs showing Giemsa-stained macrophage cells from control and treated groups (×100). (**a**) Appearance of amastigote survival in macrophages from the control group (**b**) appearance of macrophages exposed only to Glucantime (**c**) appearance of macrophages exposed only to AgNPs, and (**d**–**f**) appearance of vacuoles in macrophages infected with *L. tropica* amastigotes and treated with nanofiber formulations containing different concentrations of Glucantime; (**d**) PVA-AgNPs/Gel-Glu1.2%/PVA, (**e**) PVA-AgNPs/Gel-Glu2.4%/PVA, (**f**) PVA-AgNPs/Gel-Glu3.6%/PVA. Black arrows indicate parasite. Red arrows indicate vacuoles.

Determination of antileishmanial activity in amastigote-macrophage cultures: Further assays were conducted to assess whether nanofiber formulations that were previously effective over the promastigote form also exert inhibitory effects on amastigotes. To evaluate anti-amastigote efficacy of tested formulations a microscopic examination was employed to visualize both infected macrophages and surviving amastigotes in pursuit of treatment with nanofibers, AgNPs and Glucantime (Figure 10). The large holes observed in vacuoles of infected macrophages that were encountered with investigated formulations were predicted to appear due to inhibition of treated amastigotes (Figure 10d–f). Numerous amastigotes that were detected in macrophages indicate the survival of parasites within untreated control group. Experimental findings reveal that low-dose silver nanoparticles incorporated into nanofibers, in combination with Glucantime, hold significant potential as therapeutic candidates for cutaneous leishmaniasis since these biomaterials absolutely eliminated the emergence of amastigotes in host cells. As for the possible mechanism of action, it is suggested that silver ions released from the nanofibers suppress amastigotes by stimulating high levels of nitric oxide production, increasing ROS generation, and enhancing enzymatic activity within macrophage cells [106].

A quantitative analysis was also conducted for comparative evaluation of amastigote clearance in macrophages exposed to different nanofibers and formulations through infection index values. The obtained outcomes obviously enlightened that nanofibers incorporated with the drug and silver nanoparticles were the most influential treatment approach among tested formulations in terms of inhibition of growth, multiplication and survivability of *L. tropica* amastigotes. It was also explored that amastigote clearance ratio vigorously lifted in groups treated with nanofibers in response to the increment at Glucantime concentration within the biomaterial. Nevertheless, utilization of silver nanoparticles and Glucantime solely resulted in partial decline in amastigote numbers within host cells in comparison with nanofiber application. In the experimental groups where varying concentrations of AgNPs were applied individually, even the lowest concentration resulted in an approximately 2.3-fold reduction in amastigote numbers. A comparable reduction was observed with the lowest concentration of Glucantime applied alone. In both groups, increasing the concentration further inhibited amastigotes, leading to a >5-fold decrease in amastigote numbers compared with the control. While the infection index in the control group was 410, it was recorded as 62, 54, and 22 for Glucantime-loaded nanofibers at concentrations of 1.2, 2.4, and 3.6% (*w*/*w*), respectively. Each active compound–loaded nanofiber formulation, at its respective concentration, strongly suppressed amastigote survival within macrophages, resulting in a 6- to 18-fold reduction in infection index. Accordingly, all experimental groups demonstrated a substantial decrease in macrophage infection index and exhibited inhibitory effects against amastigotes. However, nanofibers were superior regarding the elimination of amastigotes, according to infection index results (Table 4). Furthermore, incorporation of a non-toxic concentration of AgNPs into the nanofiber enhanced the inhibitory effect on amastigotes from 1.5-fold to 18-fold, without exerting any cytotoxicity toward macrophages.

## 4. Conclusions

This study developed a novel three-layered electrospun nanofiber wound dressing incorporating green-synthesized AgNPs and Glucantime for the treatment of cutaneous leishmaniasis. Physicochemical and morphological analyses confirmed the uniform distribution, compositional integrity, and thermal stability of the fabricated nanofibrous system. The incorporation of Glucantime and AgNPs significantly influenced the nanofiber structure and surface characteristics, leading to enhanced functional performance.

Consequently, the outcomes of the current study exert that nanofiber incorporated with glucantime and AgNPs derived propolis demonstrated marvelous antileishmanial activity on both *L. tropica* promastigotes and amastigotes. It is also deciphered that inhibitory effect of synthetized nanofibers excessively augmented dependent on the elevation of Glucantime concentration within the scaffold. Another captivating result of this paper is that none of explored nanofibers stimulated cytotoxicity on host macrophages. Considering well-known drawbacks of present antileishmanial medications such as toxicity, drug resistance and intolerable side effects, overwhelming outputs of this article is very promising for further substitution between newly tailored nanofibers and disadvantageous ancient drug applications. We believe that recently generated nanofibers reinforced with Glucantime and AgNPs will open a new era for the combat against cutaneous leishmaniasis thanks to their outstanding biocompatibility feature and brilliant antileishmanial activity. While the present study was conducted in vitro, the electrospun nanofiber architecture resembles key features of the extracellular matrix, and the biocompatibility of the polymers used in this system has been well documented in the literature, suggesting that a toxic immune response is unlikely in an in vivo wound environment. In addition, the nanofiber structure may absorb wound exudate, thereby initiating material degradation and contributing to the wound healing process. This new generation, being biocompatible and highly effective, would be further utilized confidently in eradication of this tropical disease. However, in vivo examinations and clinical trials should be carried out first.

## Figures and Tables

**Table 1:** Compositions of wound dressings.

Sample Name	AgNPs % (*v*/*v*)	Glucantime % (*w*/*w*)
PVA	0	0
PVA-AgNPs	20	0
Gel	0	0
Gel-Glu3.6%	0	3.6
PVA/Gel/PVA	0	0
PVA-AgNPs/Gel/PVA	20	0
PVA-AgNPs/Gel-Glu1.2%/PVA	20	1.2
PVA-AgNPs/Gel-Glu2.4%/PVA	20	2.4
PVA-AgNPs/Gel-Glu3.6%/PVA	20	3.6

**Table 3:** Kinetic parameters obtained from different release models for Glucantime and AgNPs-loaded tri-layer composite nanofiber.

Kinetic Models	Glucantime		AgNPs		
	R^2^	k	R^2^	k	n
Zero-order	0.1141	0.0011 mg/L.h	0.819	0.0259 mg/L.h	-
First-order	0.0766	0.0002 h^−1^	0.9669	0.0007 h^−1^	-
Higuchi	0.3421	0.2847 mg/h^1/2^	0.9422	1.8666 mg/h^1/2^	-
Korsmeyer-Peppas	-	-	0.9859	0.0895 h^−n^	0.2372

**Table 4:** Infection index of amastigote-infected macrophages after treatment with nanofibers containing AgNPs, Glucantime, or their combination.

Experimental Groups	Infection Index Values
Control	410 ± 20.5
PVA/Gel/PVA	250 ± 12.5
PVA-AgNPs/Gel/PVA	204 ± 10.2
PVA-AgNPs/Gel-Glu1.2%/PVA	62 ± 3.1
PVA-AgNPs/Gel-Glu2.4%/PVA	54 ± 2.7
PVA-AgNPs/Gel-Glu3.6%/PVA	22 ± 1.1
AgNPs 25 μg/mL	174 ± 8.7
AgNPs 50 μg/mL	97 ± 4.85
AgNPs 100 μg/mL	99 ± 4.95
AgNPs 200 μg/mL	79 ± 3.95
Glucantime 50 μg/mL	175 ± 8.75
Glucantime 100 μg/mL	161 ± 8.05
Glucantime 200 μg/mL	159 ± 7.95
Glucantime 400 μg/mL	74 ± 3.7

## Data Availability

The original contributions presented in the study are included in the article, further inquiries can be directed to the corresponding author.

## Supplementary Materials

The following supporting information can be downloaded at: https://www.mdpi.com/article/10.3390/jfb17010041/s1. Figure S1. Particle size (Z-Ave) and polydispersity index (PDI) of silver nanoparticles synthesized at different pH values: (a) pH 7.5, (b) pH 9.5, and (c) pH 11.5. Figure S2. Particle size (Z-Ave) and polydispersity index (PDI) of silver nanoparticles synthesized at different temperatures: (a) 40 °C, (b) 60 °C, and (c) 80 °C. Figure S3. Particle size (Z-Ave) and polydispersity index (PDI) of silver nanoparticles synthesized at different AgNO_3_-to-extract ratios (*v*:*v*): (a) 19.5:0.5, (b) 19:1, (c) 18:2, (d) 15:5, and (e) 10:10. Figure S4. TGA and DTG thermal curves of (a) AgNPs, (b) PVA nanofiber, (c) PVA-AgNPs nanofiber, (d) Glucantime, (e) Gel nanofiber, (f) Gel-Glu3.6% nanofiber and, (g) PVA-AgNPs/Gel-Glu3.6%/PVA nanofiber. Table S1. Zeta potential (mV) of silver nanoparticles synthesized at different pH values. Table S2. Zeta potential (mV) of silver nanoparticles synthesized at different temperatures. Table S3. Zeta potential (mV) of silver nanoparticles synthesized at different AgNO_3_-to-extract ratios (*v*:*v*).
