# Supplementary material for: Tri-Layer Composite Nanofiber Wound Dressing Incorporating Glucantime and Silver Nanoparticles for Cutaneous Leishmaniasis Management
Source: J Funct Biomater. 2026 Jan 15;17(1):41. doi: 10.3390/jfb17010041 (PMC12842257; doi:10.3390/jfb17010041)
Supplement: Supplementary file 1 [file jfb-17-00041-s001.zip › jfb-4034977-supplementary.pdf]

Supporting Information

Tri-Layer Composite Nanofiber Wound Dressing Incorporating Glucantime and Silver Nanoparticles for Cutaneous Leishmaniasis Management

Hilal Topuz[1,2], Murat Inal[3], Atiye Turker[1,4], Zisan Toprak[1], Emrah Sefik Abamor[1], Sezen Canim Ates[5*], Serap ACAR[1*]

[1]Department of Bioengineering, Faculty of Chemical and Metallurgical, Yildiz Technical University, Istanbul 34210, Turkey
[2]Department of Materials and Material Processing Technologies, Kirikkale Vocational School, Kirikkale University, Kirikkale 71450, Turkey
[3]Department of Bioengineering, Faculty of Engineering and Natural Sciences, Kirikkale University, Kirikkale 71450, Turkey
[4]Department of Medical Services and Techniques, School of Vocational of Healthy, Istinye University, Istanbul 34010, Turkey
[5]Department of Biomedical Engineering, Faculty of Engineering and Architecture, Istanbul Yeni Yuzyil University, Istanbul 34010, Turkey

***Corresponding Authors:**

Prof. Dr. Serap Acar

Telephone: +90 212 383 46 43

Fax: +90 212 383 46 25

e-mail: serapacar5@gmail.com

Assoc. Prof. Sezen Canim Ates

Telephone: +90 444 5001

Fax: +90 212 481 40 58

e-mail: sezen.canimates@yeniyuzyil.edu.tr

Process Optimization for the Green Synthesis of Silver Nanoparticles

pH optimization

Nanoparticle synthesis was performed at an 18:2 $AgNO_3$/extract ratio (v:v) and room temperature with three different pH values (7.5, 9.5, and 11.5). As shown in Figure S1, the formulation at pH 9.5 yielded the most favorable particle size and PDI values.

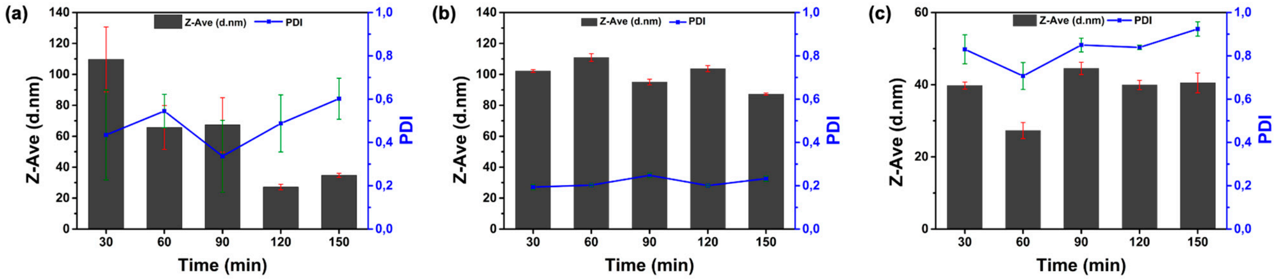

Figure S1 Particle size (Z-Ave) and polydispersity index (PDI) of silver nanoparticles synthesized at different pH values: (**a**) pH 7.5, (**b**) pH 9.5, and (**c**) pH 11.5.

Increasing the pH from 7.5 to 9.5 led to more negative zeta potential values, indicating improved colloidal stability. Zeta potential values at pH 9.5 and 11.5 were comparable, both reflecting good stability. Considering the lower PDI obtained at pH 9.5, this pH was selected as the optimum synthesis condition (Table S1). Although the synthesis reached completion at 120 min, data were recorded up to 150 min to ensure consistency across all optimization experiments.

Table S1 Zeta potential (mV) of silver nanoparticles synthesized at different pH values.

Time (min)	pH 7.5	pH 9.5	pH 11.5
30	-12.53±1.72	-17.4±0.814	-16.2±0.723
60	-12.2±4.71	-21.4±1.49	-16.2±0.723
90	-10.2±4.32	-18.4±0.757	-18.8±0.850
120	-12.9±2.40	-20.3±2.51	-17.6±0.794
150	-17.7±4.51	-15.3±2.67	-20.5±1.31

Temperature optimization

Further optimization was carried out at an 18:2 $AgNO_3$/extract ratio (v:v) and pH 9.5 under three different temperatures (40 °C, 60 °C, and 80 °C). The effect of temperature on particle size and PDI is shown in Figure S2.

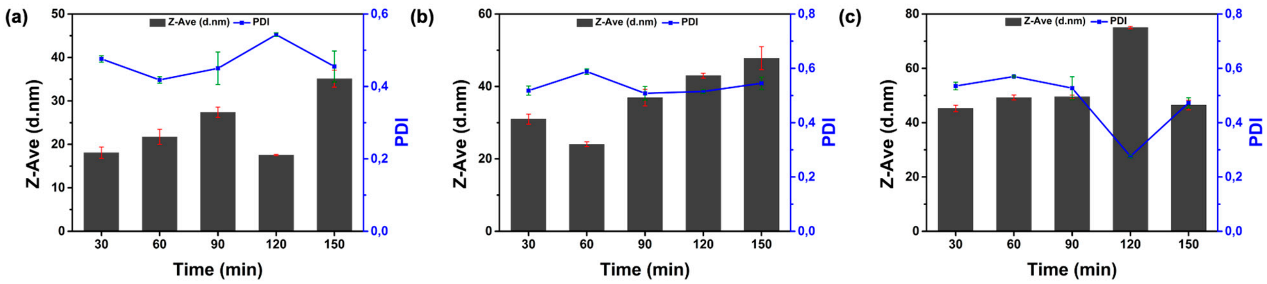

Figure S2 Particle size (Z-Ave) and polydispersity index (PDI) of silver nanoparticles synthesized at different temperatures: (**a**) 40 °C, (**b**) 60 °C, and (**c**) 80 °C.

Increasing the synthesis temperature led to more negative zeta potential values, indicating improved colloidal stability. Zeta potential values at 60 °C and 80 °C were comparable, both reflecting good stability. Considering the lower PDI obtained at 80 °C, this temperature was selected as the optimum synthesis condition (Table S2).

Table S2 Zeta potential (mV) of silver nanoparticles synthesized at different temperatures.

Time (min)	40 °C	60 °C	80 °C
30	-10.7±0.819	-12.9±0.473	-12.6±0.981
60	-10.8±1.58	-16.7±1.87	-14.3±1.27
90	-10.9±0.700	-17.9±2.07	-16±1.07
120	-10.6±0.150	-16.9±0.513	-17.1±0.820
150	-10.6±0.640	-16±0.961	-17.4±1.04

$AgNO_3$: extract ratio optimization

Different $AgNO_3$-to-extract ratios (v:v) (19.5:0.5, 19:1, 18:2, 15:5, and 10:10) were evaluated at pH 9.5 and 80 °C. As shown in Figure S3, some ratios produced slightly smaller particle sizes than the 18:2 formulation at certain reaction times; however, 18:2 exhibited the lowest PDI, indicating a more homogeneous particle distribution and better overall stability.

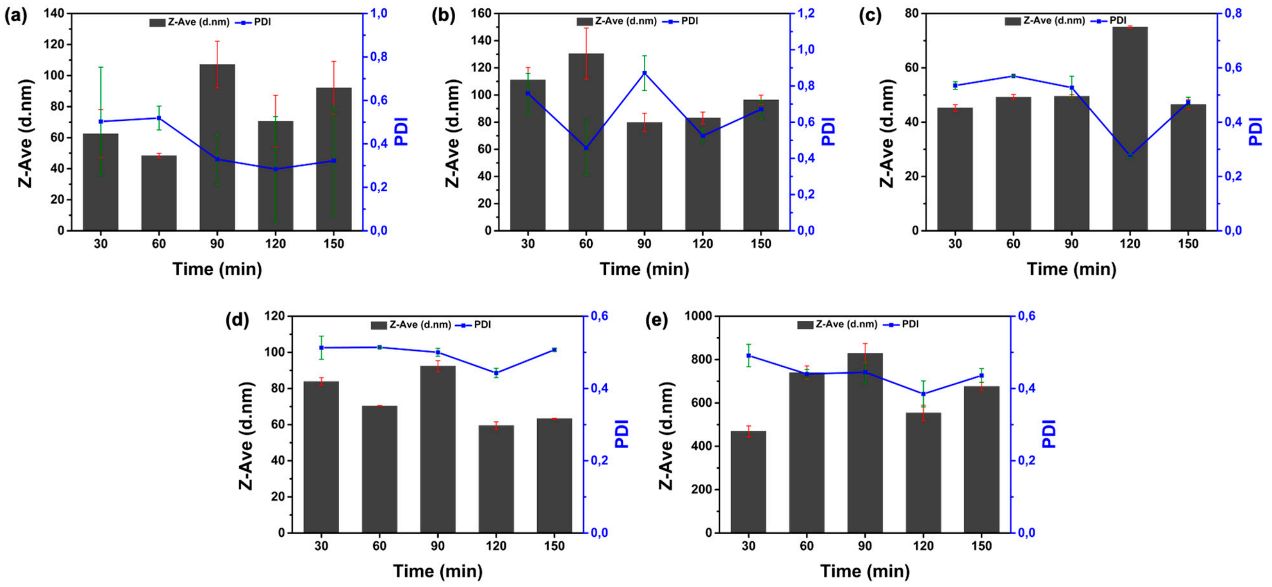

Figure S3 Particle size (Z-Ave) and polydispersity index (PDI) of silver nanoparticles synthesized at different $AgNO_3$-to-extract ratios (v:v): (**a**) 19.5:0.5, (**b**) 19:1, (**c**) 18:2, (**d**) 15:5, and (**e**) 10:10.

Zeta potential values were mostly similar among the tested ratios, while the 10:10 ratio showed a more negative value, indicating better colloidal stability. However, the particle sizes at this ratio exceeded 400 nm, which reduced its suitability (Table S3).

Table S3 Zeta potential (mV) of silver nanoparticles synthesized at different $AgNO_3$-to-extract ratios (v:v).

Time (min)	19.5:0.5	19:1	18:2	15:5	10:10
30	-19.5±5.03	-13.6±3.76	-12.6±0.981	-19.4±1.53	-24.5±0.351
60	-16.5±3.76	-11.2±0.929	-14.3±1.27	-17.4±1.29	-25.5±0.451
90	-14.7±3.11	-16±4.56	-16±1.07	-16.9±0.624	-25.5±0.400
120	-17.1±1.31	-15.6±2.74	-17.1±0.820	-17.7±0.854	-26.1±0.416
150	-20.1±1.35	-13±0.702	-17.4±1.04	-16.2±0.208	-26±0.252

Thermogravimetric and Derivative Thermogravimetric Analysis (TGA/DTG)

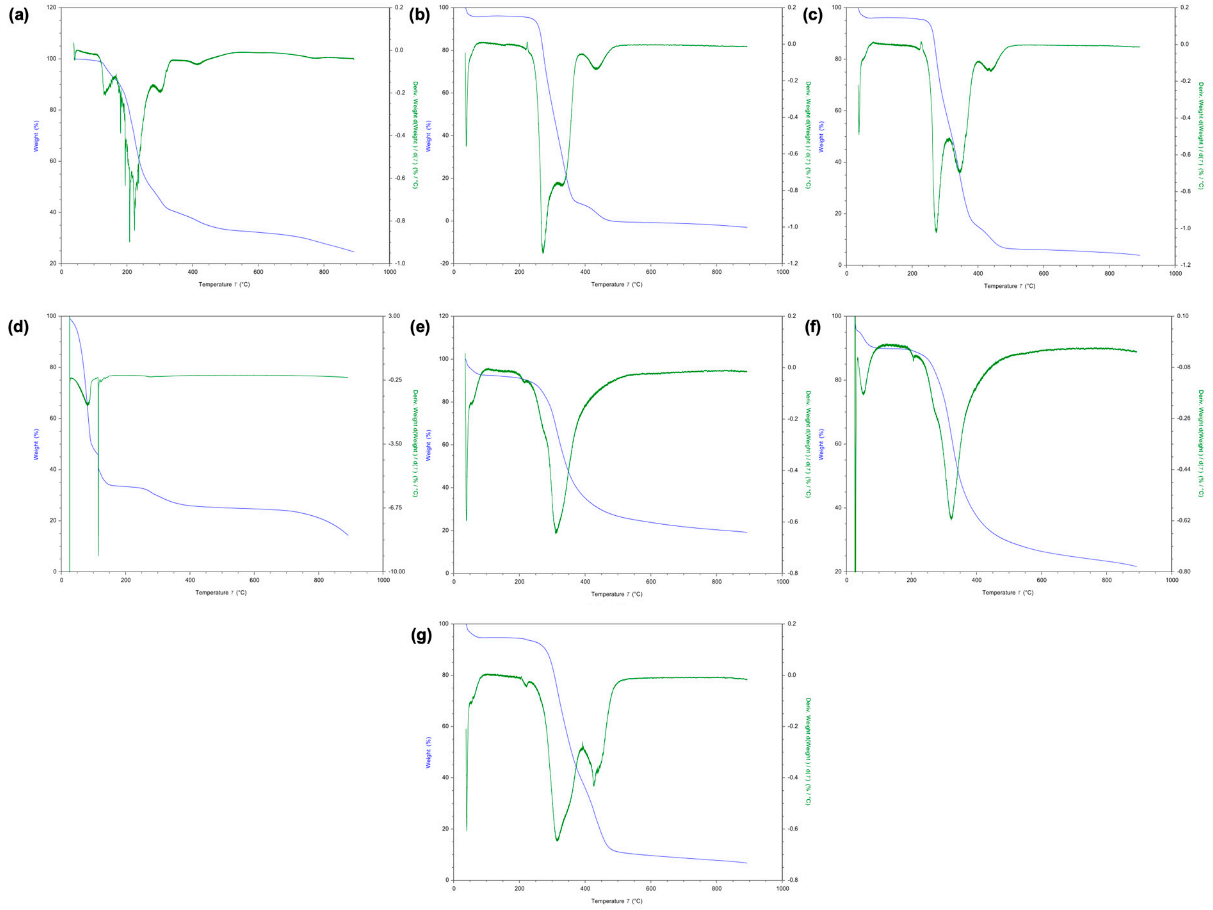

Figure S4. TGA and DTG thermal curves of **(a)** AgNPs, **(b)** PVA nanofiber, **(c)** PVA-AgNPs nanofiber, **(d)** Glucantime, **(e)** Gel nanofiber, **(f)** Gel-Glu3.6% nanofiber and, **(g)** PVA-AgNPs/Gel-Glu3.6%/PVA nanofiber.
